# Coordination Polymers Constructed from an Adaptable Pyridine-Dicarboxylic Acid Linker: Assembly, Diversity of Structures, and Catalysis

**DOI:** 10.1021/acs.inorgchem.2c01855

**Published:** 2022-11-01

**Authors:** Xiaoyan Cheng, Lirong Guo, Hongyu Wang, Jinzhong Gu, Ying Yang, Marina V. Kirillova, Alexander M. Kirillov

**Affiliations:** † State Key Laboratory of Applied Organic Chemistry, Key Laboratory of Nonferrous Metal Chemistry and Resources Utilization of Gansu Province, College of Chemistry and Chemical Engineering, Lanzhou University, Lanzhou 730000, People’s Republic of China; ‡ Centro de Química Estrutural, Institute of Molecular Sciences, Departamento de Engenharia Química, Instituto Superior Técnico, Universidade de Lisboa, Av. RoviscoPais, 1049-001 Lisbon, Portugal

## Abstract

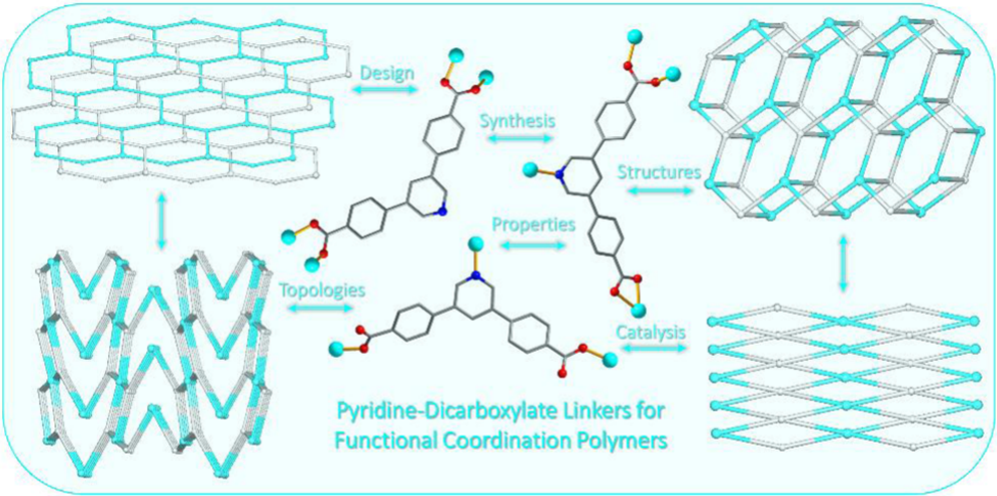

4,4′-(Pyridine-3,5-diyl)dibenzoic acid (H_2_pdba) was explored as an adaptable linker for assembling a diversity of new manganese(II), cobalt(II/III), nickel(II), and copper(II) coordination polymers (CPs): [Mn(μ_4_-pdba)(H_2_O)]_*n*_ (**1**), {[M(μ_3_-pdba)(phen)]·2H_2_O}_*n*_ (M = Co (**2**), Ni (**3**)), {[Cu_2_(μ_3_-pdba)_2_(bipy)]·2H_2_O}_*n*_ (**4**), {[Co(μ_3_-pdba)(bipy)]·2H_2_O}_*n*_ (**5**), [Co_2_(μ_3_-pdba)(μ-Hbiim)_2_(Hbiim)]_*n*_ (**6**), and [M(μ_4_-pdba)(py)]_*n*_ (M = Co (**7**), Ni (**8**)). The CPs were hydrothermally synthesized using metal(II) chloride precursors, H_2_pdba, and different coligands functioning as crystallization mediators (phen: 1,10-phenanthroline; bipy: 2,2′-bipyridine, H_2_biim: 2,2′-biimidazole; py: pyridine). Structural networks of **1**–**8** range from two-dimensional (2D) metal–organic layers (**1**–**3**, **5**–**8**) to three-dimensional (3D) metal–organic framework (MOF) (**4**) and disclose several types of topologies: **sql** (in **1**), **hcb** (in **2**, **3**, **5**), **tfk** (in **4**), 3,5L66 (in **6**), and SP 2-periodic net (6,3)Ia (in **7**, **8**). Apart from the characterization by standard methods, catalytic potential of the obtained CPs was also screened in the Knoevenagel condensation of benzaldehyde with propanedinitrile to give 2-benzylidenemalononitrile (model reaction). Several reaction parameters were optimized, and the substrate scope was explored, revealing the best catalytic performance for a 3D MOF **4**. This catalyst is recyclable and can lead to substituted dinitrile products in up to 99% product yields. The present study widens the use of H_2_pdba as a still poorly studied linker toward designing novel functional coordination polymers.

## Abstract 2

4,4′-(Pyridine-3,5-diyl)dibenzoic acid (H_2_pdba) was applied as an adaptable linker for the synthesis of new coordination polymers with varying structural multiplicity, topological types, and catalytic activity in Knoevenagel condensation. This work broadens the use of H_2_pdba as a still little investigated linker toward designing novel types of metal−organic architectures.

## Introduction

Coordination polymers (CPs) are currently recognized as highly auspicious functional compounds owing to their captivating structural features, unique properties, and extensive diversity of applications in gas separation and storage,^1−6^ sensing,^7−11^ bioactive materials,^12−14^ catalysis,^15−22^ and many other areas.^23−27^ The properties of CPs are governed by their structural and topological characteristics, as well as types of linkers and metal nodes.^28−30^ Although the synthesis of coordination polymers can be tuned toward explicit structural and topological types,^31−33^ a full control of the outcome of self-assembly reactions remains challenging because many factors can have an influence on the crystallization of CPs, namely, connectivity of linkers,^34,35^ coordination preferences of metal ions,^36,37^ presence of coligands, as well as reaction parameters (solvents, temperatures, pH values).^38−42^

Among a high diversity of linkers for the assembly of coordination polymers, aromatic polycarboxylic acids are widely applied owing to their tunable size, good stability, multiple coordination sites, and versatile coordination modes.^37−43^ Previously, we focused on designing novel CPs driven by multicarboxylate linkers incorporating diphenyl, triphenyl, or phenylpyridine cores,^19,20,44,45^ followed by the exploration of the obtained materials as catalysts in different organic transformations.

Among such reactions, Knoevenagel condensation is an important process in synthetic organic chemistry, wherein α,β-unsaturated products are generated via a nucleophilic addition between carbonyl substrates and methylene nucleophiles, followed by a dehydration step.^46−49^ Knoevenagel condensation catalyzed by different coordination compounds, including metal–organic frameworks (MOFs) and CPs, has seen a considerable development^50−53^ owing to the high efficiency and recyclability of these types of catalysts.

**Scheme 1:**
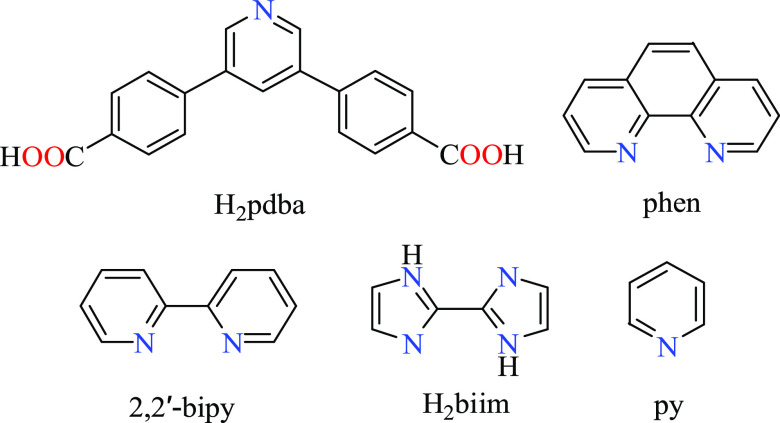
Structures of H_2_pdba and Coligands

As a continuation of our line of research devoted to the assembly of functional coordination polymers and their use in catalytic transformations, the principal aim of this work consisted in studying a pyridine-dicarboxylate building block, 4,4′-(pyridine-3,5-diyl)dibenzoic acid (H_2_pdba, Scheme 1), as a linker for the synthesis of novel CPs. The motivation to select H_2_pdba was based on several aspects: (1) presence of three aromatic rings with possible C–C bond twisting and rotation to enable adaptable angles between adjacent aromatic planes; (2) existence of mixed O- and N-coordination sites (two COOH groups and a pyridine ring); and (3) stability of H_2_pdba under hydrothermal conditions along with a still modest application of this linker for constructing CPs.^54−59^

Hence, the present work reports the hydrothermal assembly, complete characterization, X-ray structural features, topological analysis, thermal stability, and catalytic behavior of eight new coordination polymers (Scheme 1) assembled from the pdba^2–^ linkers and different coligands acting as mediators of crystallization. On the basis of elemental analysis, thermogravimetric analysis (TGA), Fourier transform infrared spectroscopy (FTIR), powder X-ray diffraction (PXRD), and single-crystal structural characterization, the isolated CPs were formulated as: [Mn(μ_4_-pdba)(H_2_O)]_*n*_ (**1**), {[M(μ_3_-pdba)(phen)]·2H_2_O}_*n*_ (M = Co (**2**), Ni (**3**)), {[Cu_2_(μ_3_-pdba)_2_(bipy)]·2H_2_O}_*n*_ (**4**), {[Co(μ_3_-pdba)(bipy)]·2H_2_O}_*n*_ (**5**), [Co_2_(μ_3_-pdba)(μ-Hbiim)_2_(Hbiim)]_*n*_ (**6**), and [M(μ_4_-pdba)(py)]_*n*_ (M = Co (**7**), Ni (**8**)). In addition to extending a number of CPs assembled from H_2_pdba, the present work also describes their catalytic application in the mild coupling of benzaldehyde with propanedinitrile to give 2-benzylidenemalononitrile (model Knoevenagel reaction).

## Experimental Section

### Brief Details on the Synthesis of **1–8**

All of the CPs were synthesized by a hydrothermal procedure (Table 1), wherein the reaction mixtures of diverse composition were kept in water at 160 °C for 72 h, with a subsequent gradual cooling (10 °C h^–1^) and crystallization steps. Full description of syntheses and characterization of each of the products **1**–**8** is provided in the Supporting Information (SI).

**Table 1:** Summary on the Hydrothermal Synthesis of CPs **1**–**8**^a^

formula of CP	metal(II) precursor	coligand (crystallization mediator: CM)	M^2+^/H_2_pdba/CM/NaOH molar ratio	network dimensionality	topology
[Mn(μ_4_-pdba)(H_2_O)]_*n*_ (**1**)	MnCl_2_·4H_2_O		1/1/-/2	2D	**sql**
{[Co(μ_3_-pdba)(phen)]·2H_2_O}_*n*_ (**2**)	CoCl_2_·6H_2_O	phen	1/1/1/2	2D + 2D^b^	**hcb**
{[Ni(μ_3_-pdba)(phen)]·2H_2_O}_*n*_ (**3**)	NiCl_2_·6H_2_O	phen	1/1/1/2	2D + 2D^b^	**hcb**
{[Cu_2_(μ_3_-pdba)_2_(2,2′-bipy)]·2H_2_O}_*n*_ (**4**)	CuCl_2_·2H_2_O	2,2′-bipy	1/1/1/2	3D	**tfk**
{[Co(μ_3_-pdba)(2,2′-bipy)]·2H_2_O}_*n*_ (**5**)	CoCl_2_·6H_2_O	2,2′-bipy	1/1/1/2	2D + 2D^b^	**hcb**
[Co_2_(μ_3_-pdba)(μ-Hbiim)_2_(Hbiim)]_*n*_ (**6**)	CoCl_2_·6H_2_O	H_2_biim	1/1/1/2	2D	3,5L66
[Co(μ_4_-pdba)(py)]_*n*_ (**7**)	CoCl_2_·6H_2_O	py	1/1/31/-	2D	SP 2-periodic net (6,3) Ia
[Ni(μ_4_-pdba)(py)]_*n*_ (**8**)	NiCl_2_·6H_2_O	py	1/1/31/-	2D	

a Hydrothermal synthesis: Teflon-lined stainless steel reactor (volume: 25 mL), H_2_O solvent (10 mL), 72 h, 160 °C.

b Two interpenetrated nets.

### Structural Characterization

Bruker Smart CCD or Agilent SuperNova diffractometers (graphite-monochromated Cu Kα radiation, λ = 1.54184 Å) were used for collecting the single-crystal X-ray data for CPs **1**–**8**. SADABS program was used for running semiempirical absorption correction. SHELXS-97 and SHELXL-97^60^ were applied for solving all of the structures by direct methods and refinement by full-matrix least-squares on *F*^2^ using. The non-H atoms were anisotropically refined by full-matrix least-squares procedures on *F*^2^. The H atoms riding at C centers were placed in computed positions with fixed isotropic thermal parameters. The hydrogen atoms bound to O or N atoms were located by difference maps and constrained to riding on the respective parent atoms. In **2**–**5**, several heavily disordered crystallization water molecules were removed by SQUEEZE in PLATON.^61^ The number of lattice water molecules was estimated using the data of elemental and TGA analyses. The crystal data for **1**–**8** are summarized in Table 1. The selected bond lengths and H-bonding data are listed in Tables S1 and S2, respectively (SI).

Topos software was used for the topological classification of coordination polymers, using the concept of underlying nets. These nets were generated after removing the terminal ligands and reducing the linkers to respective centroids while preserving the connectivity.^62,63^ Supplementary crystallographic data for **1**–**8** are present in CCDC-2167498–2167505.

### Catalytic Studies (Knoevenagel Reaction)

In a typical test, the suspension containing catalyst (2 mol %; after drying and grinding), benzaldehyde (0.50 mmol), propanedinitrile (1.0 mmol), and solvent (1.0 mL, typically methanol or water) was stirred at ambient temperature for a certain reaction time. Next, the catalyst was removed using a centrifuge. Evaporation of the solvent from the filtrate under reduced pressure led to a crude solid product, which was dissolved in CDCl_3_ and subjected to ^1^H NMR spectroscopy analysis to quantify the formed product (Figure S4, SI). For performing catalyst recycling tests, the catalyst was isolated by centrifugation, rinsed with methanol several times, dried at room temperature, and reused in the next steps following the above-mentioned general procedure. Different aldehyde substrates were screened to evaluate the substrate scope. A number of blank tests were also done, revealing a significantly lower catalytic activity and/or product yields if compared to that of coordination polymers.

## Results and Discussion

### Preparation of CPs **1–8** under Hydrothermal Conditions

**Scheme 2:**
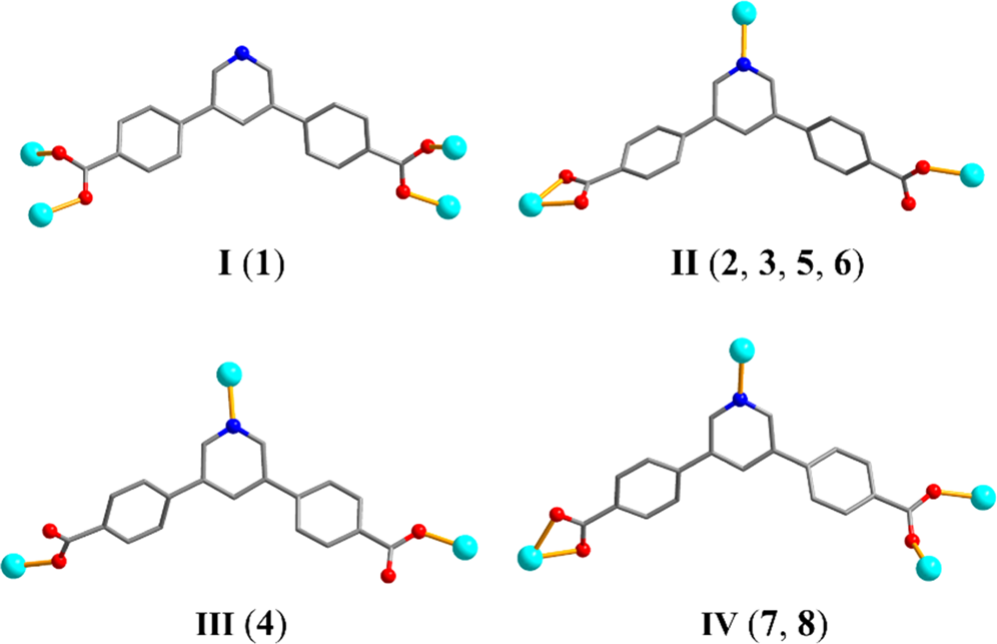
Coordination Modes (I–IV) of pdba^2–^ Linkers in **1**–**8**

To probe 4,4′-(pyridine-3,5-diyl)dibenzoic acid (H_2_pdba) as a pyridine-dicarboxylate linker for assembling different CPs based on Mn, Co, Ni, or Cu, we explored a considerable number of reactions under hydrothermal conditions (Table S3, SI), using the mixtures of metal(II) chlorides with H_2_pdba and crystallization mediators (coligands). The latter were selected from 1,10-phenanthroline (phen), 2,2′-bipyridine (bipy), 2,2′-biimidazole (H_2_biim), or pyridine (py) on the basis of their commercial availability, low cost, good stability under hydrothermal conditions, and a well-recognized role to act as coligands to mediate the crystallization of coordination polymers.^19,20,22^ Besides, when selecting these coligands, attention was paid to explore three different groups acting as potential chelators (phen, bipy), linkers (4,4′-bipy, H_2_biim), and terminal ligands (py, H_2_biim). Although 36 reaction attempts have been performed, the positive results that allowed the full characterization of products (including structural analysis) were obtained in eight cases (Scheme 2). It should be mentioned that in the absence of crystallization mediator (coligand), only compound **1** was isolated. This fact highlights the importance of coligand for facilitating the crystallization of products. The crystal structures of new CPs **1**–**8** (Table 1) were determined by single-crystal X-ray diffraction and supported by standard characterization that included FTIR, elemental analysis, TGA, and PXRD. The pyridine-dicarboxylate linkers present in **1**–**8** were originated H_2_pdba during the hydrothermal synthesis. There are four distinct coordination modes of pdba^2–^ ligands (Scheme 2) that act as μ_3_- or μ_4_-linkers. The COO^–^ groups of pdba^2–^ can adopt monodentate, bidentate, and bridging bidentate modes, while the N site of pyridine moiety is also involved in coordination in all compounds except **1**. Interestingly, the CPs **4** and **5** were obtained using the same reaction conditions but different metal(II) precursors (CuCl_2_·2H_2_O for **4** vs CoCl_2_·6H_2_O for **5**), resulting in distinct structures that are likely guided by coordination properties of the metal ions present in the reaction system. Compounds **5** and **6** were prepared using equal conditions but distinct mediators of crystallization (bipy for **5** vs H_2_biim for **6**), so their structural alterations are driven by the type of N-donor coligand. Interestingly, there is a partial oxidation of cobalt(II) ions during the synthesis of **6** that represents a Co(II)/Co(III) mixed-valence coordination polymer. In summary, the nature of metal ions and coligands eventually guides the structural and topological types of the generated CPs. However, rationalization of synthetic outcomes when using different combinations of metal ions and coligands is still elusive.

### Structural Description

#### [Mn(μ_4_-pdba)(H_2_O)]*_n_* (**1**)

**Figure 1:**
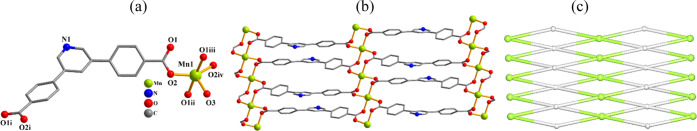
Structural fragments of **1**. (a) Coordination environment around the Mn(II) center; H atoms are omitted. (b) 2D metal–organic network; view along the *b* axis. (c) Topological representation of a mononodal 4-connected layer with a **sql** topology; view along the *b* axis; centroids of 4-connected μ_4_-pdba^2–^ nodes (gray); 4-connected Mn1 nodes (light green).

Compound **1** is a two-dimensional (2D) CP (Figure 1) having an asymmetric unit that bears one manganese atom (with half occupancy), a half of μ_4_-pdba^2–^ linker, and a half of H_2_O ligand. The Mn(II) center is five-coordinate and possesses a distorted trigonal bipyramidal {MnO_5_} environment that is constructed from four O donors from four μ_4_-pdba^2–^ linkers and one water ligand (Figure 1a). The pdba^2–^ block exhibits a μ_4_-coordination mode (mode I, Scheme 2), wherein both COO^–^ moieties are bridging bidentate while the nitrogen site of the central ring remains uncoordinated. The carboxylate groups from two μ_4_-pdba^2–^ ligands bridge the Mn1 nodes to give a chain-like motif with a metal···metal separation of 4.291(3) Å (Figure 1b). These motifs are further joined via the remaining carboxylate functionalities of μ_4_-pdba^2–^ to form a 2D metal–organic layer (Figure 1b). If considering the topology, this layer is built from the 4-linked Mn1 and μ_4_-pdba^2–^ nodes that form a mononodal 4-connected net with a (4^4^.6^2^)point symbol and **sql** topology (Figure 1c). It is necessary to note that a Zn(II) analogue of compound **1** was reported previously.^64^

#### {[Co(μ_3_-pdba)(phen)]·2H_2_O}*_n_* (**2**) and {[Ni(μ_3_-pdba)(phen)]·2H_2_O}*_n_* (**3**)

**Figure 2:**
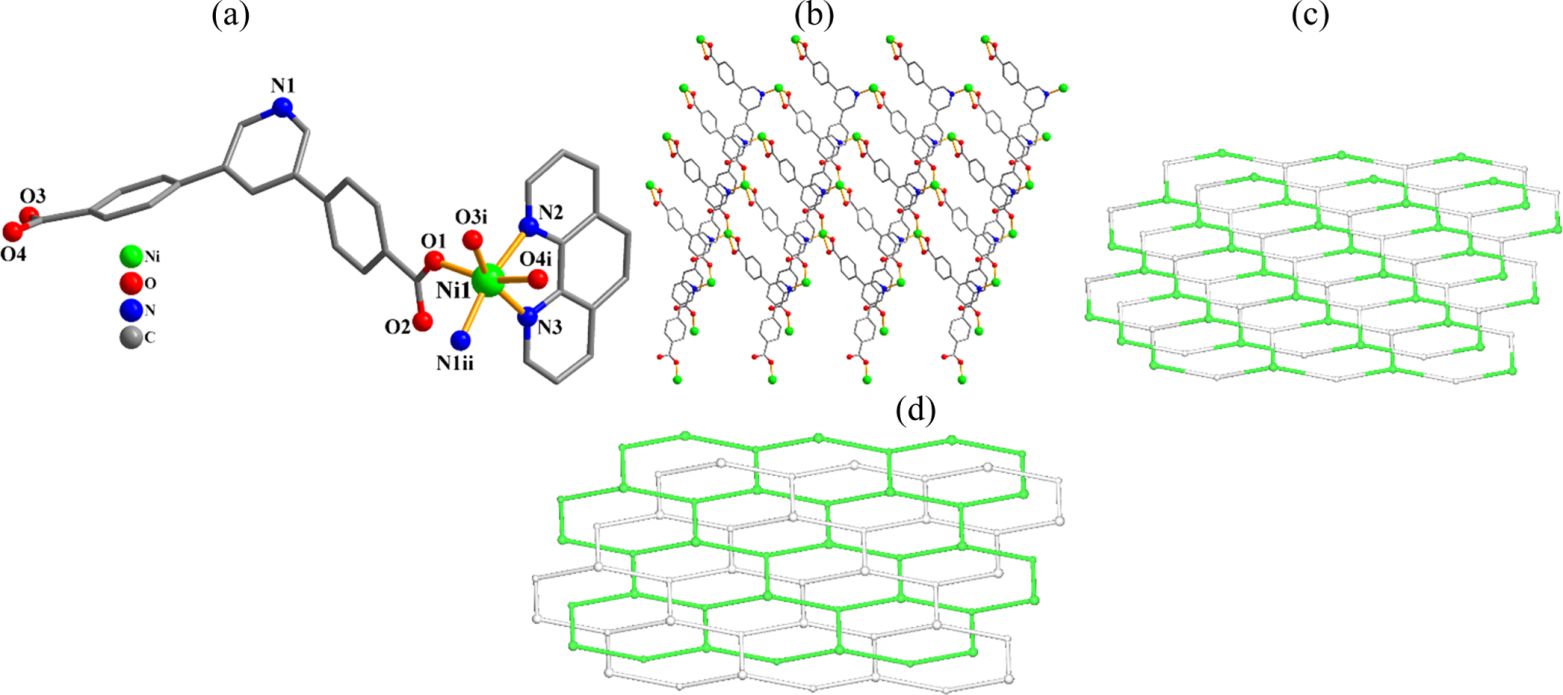
Structural fragments of **3**. (a) Coordination environment around the Ni(II) atom; H atoms are omitted. (b) 2D metal–organic network; view along the *b* axis; phen ligands are omitted. (c) Topological representation of a uninodal 3-connected metal–organic layer with a **hcb** topology; view along the *b* axis; centroids of 3-connected μ_3_-pdba^2–^ nodes (gray), 3-connected Ni1 nodes (green). (d) Two 2D + 2D interpenetrated **hcb** layers shown by different colors (green and gray).

For these isostructural CPs, the structural features of compound **3** are discussed (Figure 2). Per asymmetric entity, there is a nickel(II) atom, a μ_3_-pdba^2–^ linker, a phen moiety, and two solvent H_2_O molecules. The Ni1 center is six-coordinate and displays a distorted octahedral {NiN_3_O_3_} environment (Figure 2a), which is filled by three oxygen atoms and one N atom of three μ_3_-pdba^2–^ linkers, as well as two N_phen_ donors. The pdba^2–^ linker represents a μ_3_-coordination manner (Scheme 2, mode II), with monodentate and bidentate carboxylate groups and the coordinated pyridine N site. The Ni1 centers are linked by μ_3_-pdba^2–^ to form a 2D metal–organic network (Figure 2b). From a topological viewpoint, this network bears the 3-linked Ni1 and μ_3_-pdba^2–^ nodes (topologically equivalent) that are arranged into a mononodal 3-linked layer with a (6^3^) point symbol and **hcb** topology (Figure 2c). There are also two parallel 2D + 2D interpenetrated layers in the crystal structure which represent a notable feature of this compound (Figure 2d).

#### {[Cu_2_(μ_3_-pdba)_2_(bipy)]·2H_2_O}*_n_* (**4**)

**Figure 3:**
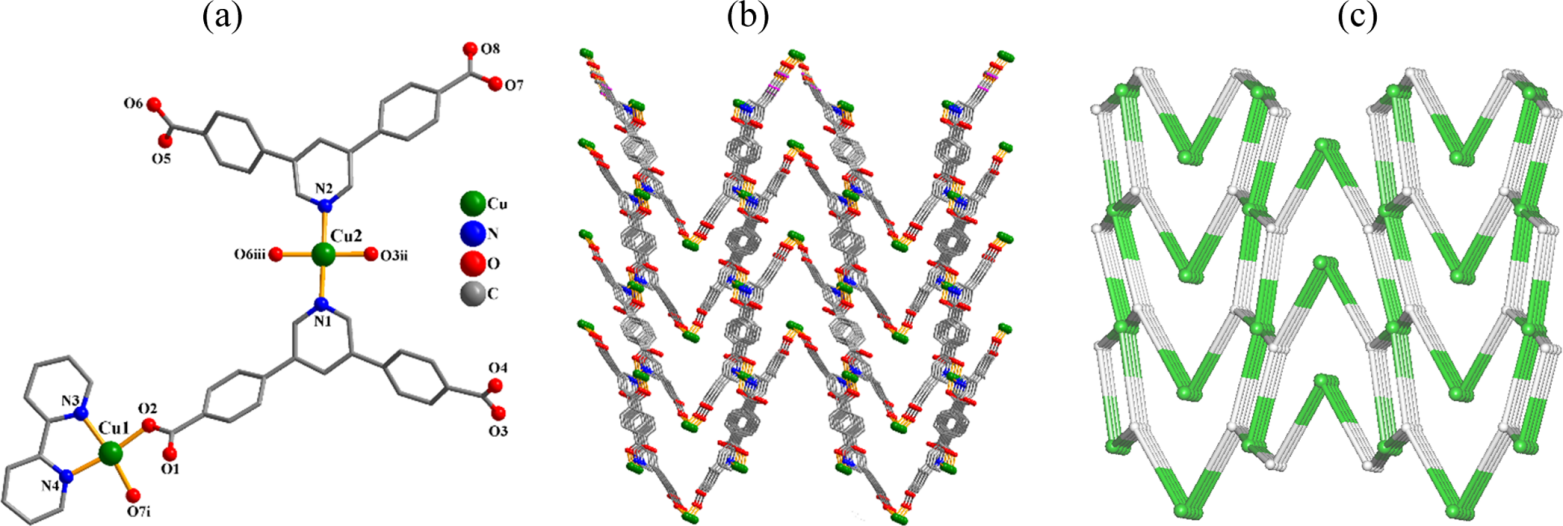
Structural fragments of **4**. (a) Coordination environment around the Cu(II) atoms; H atoms and H_2_O moieties are omitted. (b) 3D metal–organic framework; view along the *a* axis; 2,2′-bipy ligands are omitted. (c) Topological representation of a binodal 3,4-linked metal–organic framework with a **tfk** topology; view along the *a* axis; centroids of 3-connected μ_3_-pdba^2–^ nodes (gray), 2- and 4-connected Cu1 centers (green).

This structure reveals a three-dimensional (3D) metal–organic framework (Figure 3). Its asymmetric entity comprises two copper(II) centers (Cu1, Cu2), two μ_3_-pdba^2–^ linkers, one bipy moiety, and a pair of crystallization H_2_O molecules (Figure 3a). The 4-coordinate Cu1 atom displays a distorted square planar {CuN_2_O_2_} environment, which is formed by two carboxylate oxygen sites from two μ_3_-pdba^2–^ ligands and two N_bipy_ atoms. There are also two crystallization H_2_O molecules in some proximity to Cu1 (Cu1···O_water_ separations of ∼2.56 and ∼2.90 Å), which may enable some weak interactions with copper and alteration of its environment to {CuN_2_O_4_}. The Cu2 center is 4-coordinate and assumes a distorted square planar {CuN_2_O_2_} arrangement, based on two carboxylate oxygen atoms and two nitrogen donors from four μ_3_-pdba^2–^ linkers. In contrast to Cu1, there are no any potentially coordinating moieties in the proximity of Cu2. In **4**, pdba^2–^ functions as a μ_3_-linker (Scheme 2, mode III), with N-bound pyridine functionality and monodentate carboxylate groups, which are all responsible for the assembly of a 3D MOF structure (Figure 3b). Topologically, this structure is constructed from the 4-linked Cu2 and 2-linked Cu1 atoms, and the 3-linked μ_3_-pdba^2–^ nodes (Figure 3c). The resultant network can be classified as a dinodal 3,4-connected framework with a point symbol of (4·1212^2^)_2_(4^2^·12^4^) and **tfk** topology.

#### {[Co(μ_3_-pdba)(bipy)]·2H_2_O}*_n_* (**5**)

**Figure 4:**
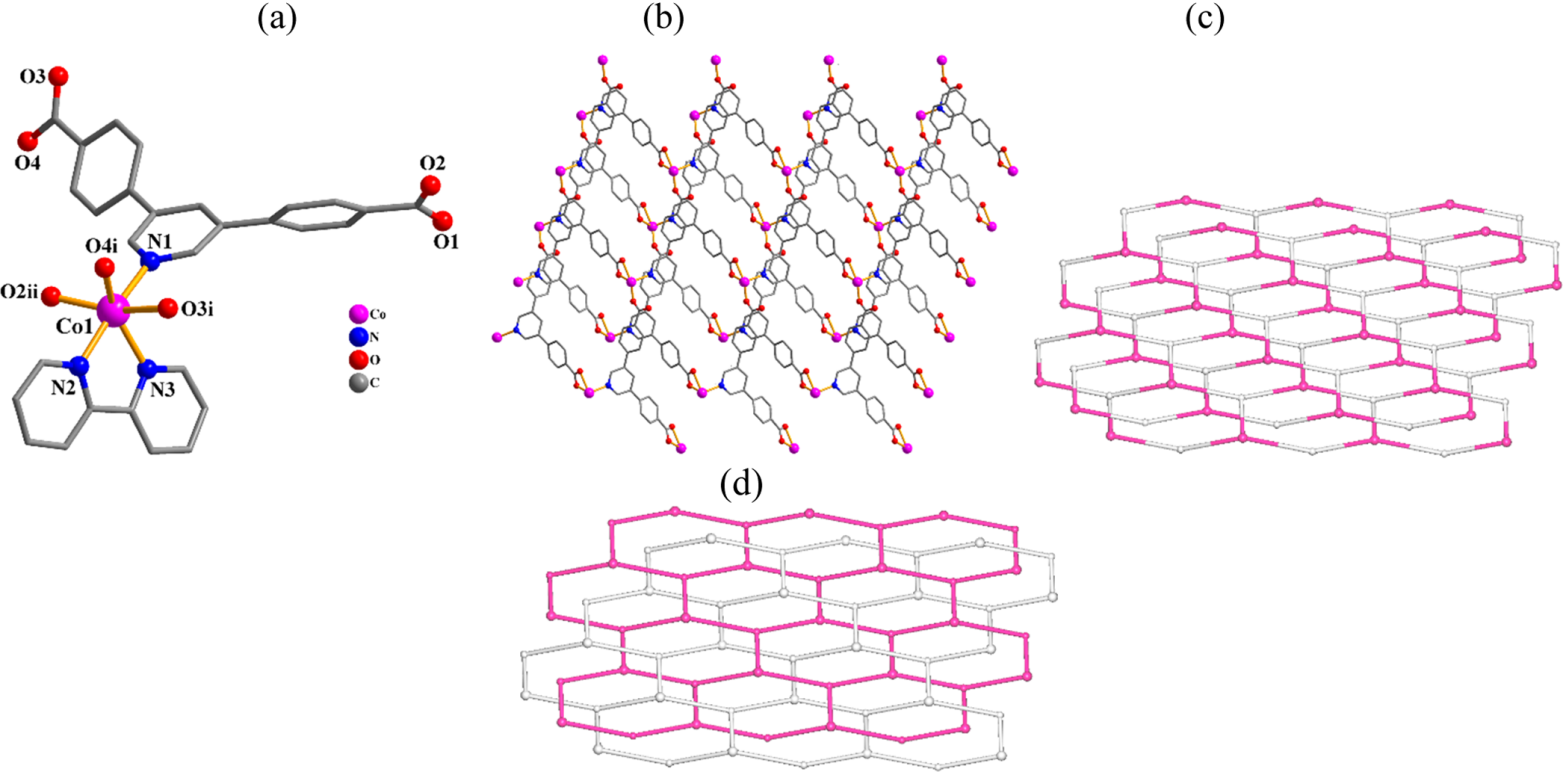
Structural fragments of **5**. (a) Coordination environment around the Co(II) atom; H atoms are omitted. (b) 2D metal–organic layer seen along the *b* axis. (c) Topological representation of a mononodal 3-linked layer with a **hcb** topology; view along the *b* axis; centroids of 3-connected μ_3_-pdba^2–^ nodes (gray), 3-connected Co1 nodes (purple). (d) Two 2D +2D interpenetrated **hcb** layers shown by different colors (purple and gray).

Compound **5** is a two-dimensional CP (Figure 4) and its asymmetric entity reveals a Co(II) atom, a μ_3_-pdba^2–^ linker, a 2,2′-bipyridine ligand, and a pair of crystallization water molecules. The Co1 center is 6-coordinate and assumes an octahedral {CoN_3_O_3_} geometrical arrangement with some distortions, which is formed by three oxygen and one nitrogen atoms from three μ_3_-pdba^2–^ linkers, along with two N_bipy_ donors (Figure 4a). In **5**, pdba^2–^ functions as a μ_3_-linker (Scheme 2, mode II) with monodentate and bidentate carboxylate groups and N-bound pyridine functionality. The μ_3_-pdba^2–^ linkers are responsible for generating a 2D layer (Figure 4b). Regarding the topological classification, this layer comprises the 3-linked Co1 and μ_3_-pdba^2–^ nodes (topologically equivalent) that are arranged into a mononodal 3-linked network with a (6^3^) point symbol and **hcb** topology (Figure 4c). This compound also features a twofold parallel 2D + 2D interpenetration (Figure 4d).

#### [Co_2_(μ_3_-pdba)(μ-Hbiim)_2_(Hbiim)]*_n_* (**6**)

**Figure 5:**
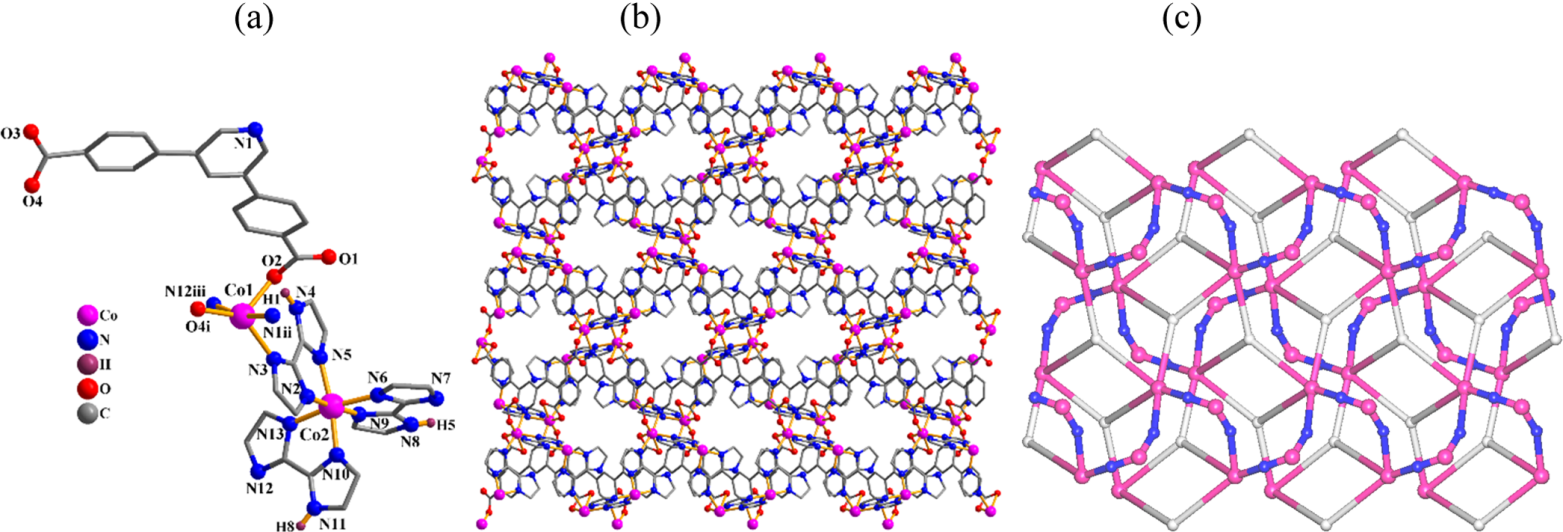
Structural fragments of **6**. (a) Coordination environment around the Co(II) centers; H atoms are omitted except in NH groups. (b) 2D metal–organic network seen along the *bc* plane. (c) Topological representation of a dinodal 3,5-linked 2D layer with a 3,5L66 topology; view along the *a* axis; centroids of 3-connected μ_3_-pdba^2–^ nodes (gray), centroids of 2-connected μ-Hbiim^–^ linkers (blue), 5-connected Co1 and 2-connected Co2 centers (purple).

Compound **6** is a mixed-valence 2D coordination polymer. The oxidation states of Co atoms in this compound were assessed by X-ray photoelectron spectroscopy (XPS; Figure S3, SI). The fitted peaks can be attributed to Co^3+^ and Co^2+^, implying the existence of cobalt centers with different oxidation states (Figure S3).^65,66^ The integrated area of Co^3+^ peaks is close to that of Co^2+^, indicating that the Co(II)/Co(III) ratio in the crystal lattice is nearly 1:1. The asymmetric unit of this coordination polymer (Figure 5) contains two distinct Co(II) and Co(III) atoms (Co1, Co2), one μ_3_-pdba^2–^ linker, two μ-Hbiim^–^ linkers, and one terminal Hbiim^–^ moiety. The 5-coordinate Co1 center reveals a distorted trigonal bipyramidal {CoN_3_O_2_} geometry, formed by two oxygen atoms and one nitrogen atom from three μ_3_-pdba^2–^ blocks and two nitrogen donors from two μ-Hbiim^–^ moieties (Figure 5a). The 6-coordinate Co2 center represents a distorted octahedral{CoN_6_} environment that is composed of four N donors from two μ-Hbiim^2–^ linkers and two nitrogen atoms from the terminal Hbiim^–^ ligand. The Co(II)–N bond lengths in compound **6** are in the range of 2.086(4)–2.273(4) Å, which are elongated in comparison with the Co(III)–N bonds varying from 1.916(4) to 1.940(4) Å. The pdba^2–^ block acts as a μ_3_-linker (Scheme 2, mode II). The bridging μ_3_-pdba^2–^ and μ-Hbiim^–^ ligands multiply interconnect the Co(II) and Co(III) centers to generate a 2D metal–organic network (Figure 5b). Topologically, the 2D layer is composed of the 5-linked Co1 and 2-linked Co2 centers, the 3-connected μ_3_-pdba^2–^ nodes, and the 2-linked μ-Hbiim^–^ ligands (Figure 5c). Such a network can be described as a dinodal 3,5-connected layer with a (4.5·6)(4.5^5^·6^3^·7) point symbol and 3,5L66 topology.

#### [Co(μ_4_-pdba)(py)]*_n_* (**7**) and [Ni(μ_4_-pdba)(py)]*_n_* (**8**)

**Figure 6:**
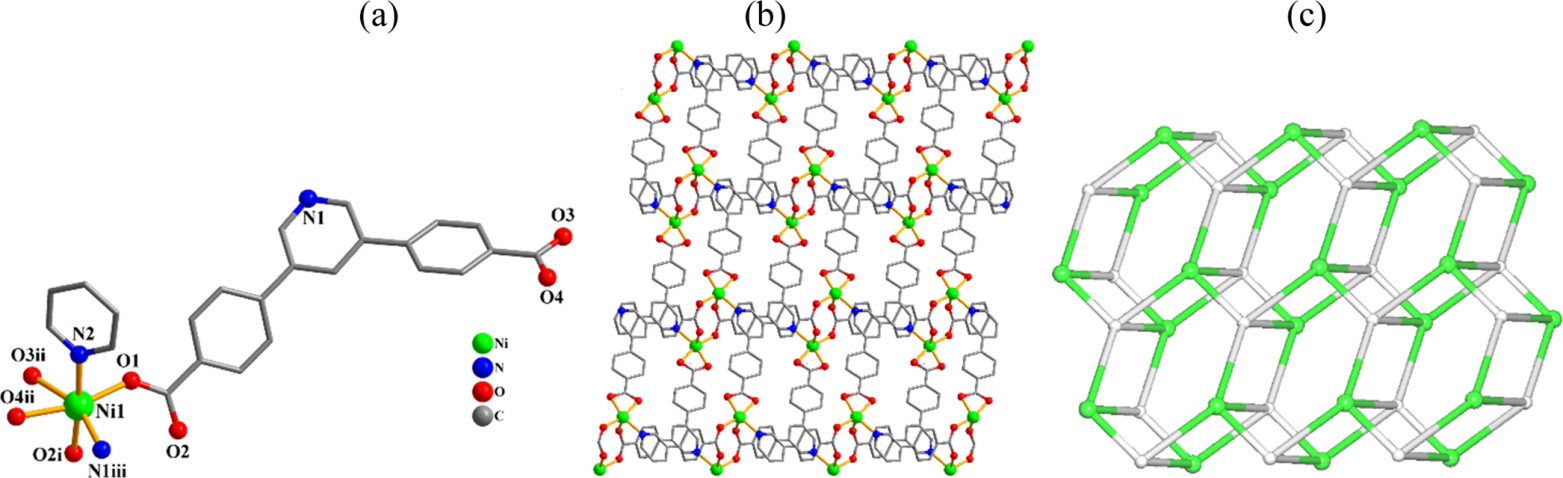
Structural fragments of **8**. (a) Coordination environment around the Ni(II) center; H atoms are omitted. (b) 2D metal–organic layer seen along the *a* axis; py ligands are omitted. (c) Topological representation of a mononodal 4-connected 2D layer with an SP 2-periodic net (6,3) Ia topology; view along the *c* axis; centroids of 4-connected μ_4_-pdba^2–^ nodes (gray); 4-connected Ni1 nodes (green).

These CPs are isomorphous (Table 2) and the structure of a 2D coordination polymer **8** is discussed as an example (Figure 6). In the asymmetric entity, there is a nickel(II) center, a μ_4_-pdba^2–^ linker, and a pyridine ligand (Figure 6a). The 6-coordinate Ni1 atoms display an octahedral {NiN_2_O_4_} geometry with distortions. It is built from four oxygen atoms and one nitrogen atom coming from four μ_4_-pdba^2–^ linkers, as well as one N_py_ donor. The pdba^2–^ ligands act as μ_4_-linkers (mode IV, Scheme 2), which interconnect two neighboring Ni1 centers into Ni_2_ subunits and then further bridge them into 2D metal–organic layers (Figure 6b). Topologically, these layers are constructed from the 4-linked Ni1 and μ_4_-pdba^2–^ nodes that generate a mononodal 4-linked net with a (4^3^·6^3^) point symbol and SP 2-periodic net (6,3)Ia topology (Figure 6c).

**Table 2:** Crystal Data for Compounds **1**–**8**

compound	**1**	**2**	**3**	**4**
chemical formula	C_19_H_13_MnNO_5_	C_31_H_23_CoN_3_O_6_	C_31_H_23_NiN_3_O_6_	C_48_H_34_Cu_2_N_4_O_10_
formula weight	390.24	592.42	592.20	953.87
crystal system	monoclinic	monoclinic	monoclinic	monoclinic
space group	*P*2/*c*	**P**2_1_/**c**	**P**2_1_/**c**	**P**2_1_/**c**
*a* (Å)	16.9176(4)	11.1536(6)	10.5968(15)	7.84272(13)
*b* (Å)	6.56360(10)	23.6165(9)	25.856(4)	20.8527(3)
*c* (Å)	7.1074(2)	11.4881(6)	11.4979(9)	24.1081(4)
α (deg)	90	90	90	90
β (deg)	100.115(2)	110.489(6)	109.127(11)	92.8058(15)
γ (deg)	90	90	90	90
*V* (Å^3^)	776.94(3)	2834.6(3)	2976.4(7)	3937.96(11)
*T* (K)	293(2)	293(2)	293(2)	293(2)
*Z*	2	4	4	4
*D*_c_ (g cm^–3^)	1.668	1.304	1.241	1.548
μ (mm^–1^)	7.210	5.071	1.249	1.869
*F*(000)	398	1140	1144	1872
Refl. measured	4517	16576	22401	24681
Unique refl. (*R*_int_)	1365 (0.0255)	5100 (0.0817)	5972 (0.1148)	7072 (0.0812)
GOF on *F*^2^	1.068	1.037	0.956	1.051
*R*_1_ [*I* > 2σ(*I*)]^a^	0.0311	0.0784	0.0776	0.0600
*w*R**_2_ [*I* > 2*σ*(*I*)]^b^	0.0886	0.2027	0.1527	0.1713

compound	**5**	**6**	**7**	**8**
chemical formula	C_29_H_23_CoN_3_O_6_	C_37_H_26_Co_2_N_13_O_4_	C_24_H_16_CoN_2_O_4_	C_24_H_16_NiN_2_O_4_
formula weight	568.40	834.57	455.32	455.10
crystal system	monoclinic	monoclinic	triclinic	triclinic
space group	**P**2_1_/**c**	**P**2_1_/*c*	*P*1̅	*P*1̅
*a* (Å)	11.0318(5)	17.9291(11)	10.3892(2)	10.3771(2)
*b* (Å)	23.5217(10)	16.1473(6)	10.9501(2)	10.8446(2)
*c* (Å)	11.5646(5)	12.4420(6)	11.0357(2)	11.0734(2)
α (deg)	90	90	65.300(2)	65.188(2)
β (deg)	110.866(5)	103.880(6)	62.921(2)	62.395(2)
γ (deg)	90	90	86.6310(10)	86.519(2)
*V* (Å^3^)	2804.1(2)	3496.9(3)	1002.06(4)	988.40(4)
*T* (K)	293(2)	293(2)	293(2)	293(2)
*Z*	4	4	2	2
*D*_c_ (g cm^–3^)	1.261	1.587	1.509	1.529
μ (mm^–1^)	5.100	7.962	7.012	1.718
*F*(000)	1092	1700	466	468
Refl. measured	10368	21279	11905	11048
Unique refl. (*R*_int_)	4870 (0.0484)	6006 (0.0742)	3720 (0.0368)	3665 (0.0313)
GOF on *F*^2^	1.029	1.088	1.058	1.084
*R*_1_ [*I* > 2σ(*I*)]^a^	0.0562	0.0649	0.0338	0.0343
*w*R**_2_ [*I* > 2σ(*I*)]^b^	0.1299	0.1244	0.0897	0.0969

a *R*_1_ = ∑||*F*_o_| – |*F*_c_||/ |*F*_o_|.

b *w*R**_2_ = {∑[*w*(*F*_o_^2^ – *F*_c_^2^)]^2^/∑[*w*(*F*_o_^2^)]^2^}^1/2^.

### Additional Characterization of **1–8**

The samples of **1**–**8** were investigated by PXRD (Figure S2, SI), revealing a good match of the experimental patterns with the simulated diffractograms that were obtained from CIF files (single-crystal data). This confirms the phase purity of the synthesized products. As some catalytic reactions were performed in water, the stability of the obtained coordination polymers was studied using the samples of CPs after being kept for 12 h in water in air at 50 °C. The PXRD patterns of dried samples after water treatment confirm that the structures of **1**–**8** are maintained (Figure S2, SI).

**Figure 7:**
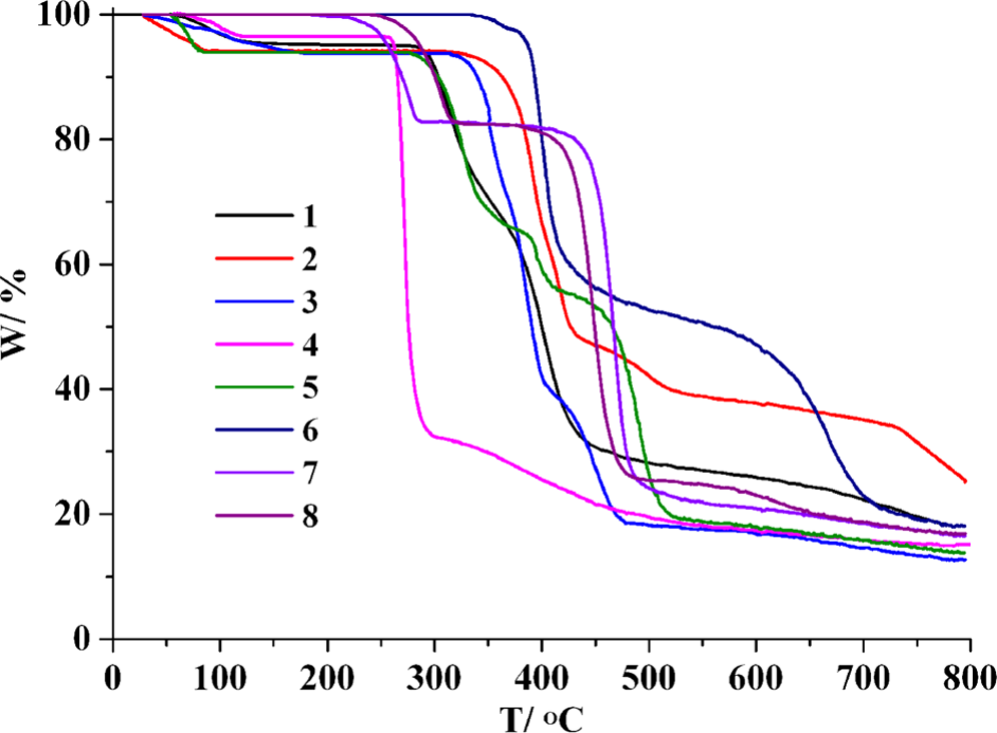
TGA curves of CPs **1**–**8**.

TGA was used to investigate the thermal stability of **1**–**8** in the 20–800 °C interval under nitrogen flow (Figure 7). For **1**, there is a loss of one water ligand at 50–128 °C (calcd, 4.6%; exptl, 4.8%) and the decomposition of the dehydrated solid starts only at 280 °C. In the case of compound **2**, a weight decrease between 30 and 84 °C corresponds to a loss of two H_2_O moieties (calcd, 6.1%; exptl, 5.8%); the dehydrated solid begins to decompose from 310 °C. The TGA of **3** reveals a mass loss (calcd, 6.1%; exptl, 6.2%) in the 28–171 °C interval corresponding to the release of two crystallization H_2_O; the dehydrated solid maintains its stability until 313 °C. For **4**, a loss of mass at 60–116 °C corresponds to a removal of two lattice water molecules (calcd, 3.4%; exptl, 3.7%), followed by the decomposition of the dehydrated compound above 255 °C. In **5**, two lattice H_2_O are released between 53 and 82 °C (calcd, 6.3%; exptl, 6.0%), and the dehydrated solid begins to decompose starting from 274 °C. There are no water ligands or solvent molecules in **6** which only decomposes above 332 °C. The TGA of **7** shows a loss of weight in the 215–287 °C range owing to the elimination of one pyridine ligand (calcd, 17.4%; exptl, 17.3%); further decomposition of the compound begins then at 391 °C. In the case of **8**, there is a mass decrease at 243–325 °C that is associated with a release of a py ligand (calcd, 17.4%; exptl, 17.6%); further decomposition of the formed sample begins only at 375 °C.

Compound **4** was selected as the most promising example in terms of catalytic performance to access its gas sorption and porosity (Table S4, SI). PLATON software was used to calculate the effective void volume of the sample without guest H_2_O molecules (5.0%). The gas sorption study was performed on an activated sample (after removal of all crystallization water) by collecting at 77 K the nitrogen adsorption/desorption isotherms. At 77 K and 1 atm, the adsorbed amount of nitrogen is 2.1 cm^3^ g^–1^. The BET surface area is 1.0 m^2^ g^–1^. The obtained data confirm a low porosity of compound **4**.

### Catalytic Knoevenagel Condensation

**Scheme 3:**
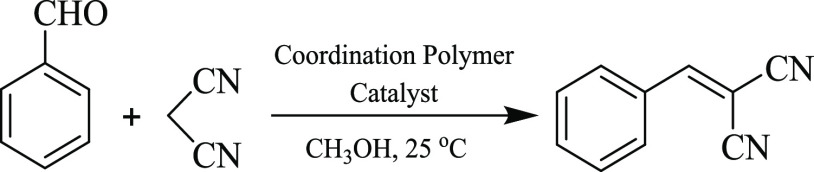
Model Condensation Reaction between Benzaldehyde and Propanedinitrile (Knoevenagel Reaction)

Considering a recognized application of coordination polymers and derivatives as catalysts in different organic transformations,^16,67−71^ we probed the obtained CPs **1**–**8** as prospective heterogeneous catalysts for the Knoevenagel condensation. Benzaldehyde was selected as a simple model substrate for the reaction with propanedinitrile to form 2-benzylidenemalononitrile (Scheme 3). Preliminary screening of all of the obtained CPs revealed that compound **4** is the most promising, which was thus explored in more detail by studying the influence of the solvent type, time of reaction, loading of catalyst, and recycling, as well as the substrate scope (Table 3).

**Table 3:** Condensation Reaction between Benzaldehyde and Propanedinitrile (Knoevenagel Reaction)^a^

entry	catalyst	temperature (°C)	time (min)	catalyst loading (mol %)	solvent	yield^b^ (%)
1	**4**	25	10	2.0	CH_3_OH	43
2	**4**	25	20	2.0	CH_3_OH	57
3	**4**	25	30	2.0	CH_3_OH	68
4	**4**	25	40	2.0	CH_3_OH	81
5	**4**	25	50	2.0	CH_3_OH	92
6	**4**	25	60	2.0	CH_3_OH	>99
7	**4**	25	60	1.0	CH_3_OH	93
8	**4**	25	60	2.0	H_2_O	98
9	**4**	25	60	2.0	C_2_H_5_OH	96
10	**4**	25	60	2.0	CH_3_CN	87
11	**4**	25	60	2.0	CHCl_3_	67
12	**1**	25	60	2.0	CH_3_OH	98
13	**2**	25	60	2.0	CH_3_OH	93
14	**3**	25	60	2.0	CH_3_OH	90
15	**5**	25	60	2.0	CH_3_OH	94
16	**6**	25	60	2.0	CH_3_OH	95
17	**7**	25	60	2.0	CH_3_OH	93
18	**8**	25	60	2.0	CH_3_OH	91
19	blank	25	60		CH_3_OH	22
20	CuCl_2_	25	60	2.0	CH_3_OH	35
21	H_2_pdba	25	60	2.0	CH_3_OH	28

a Reaction conditions: propanedinitrile (1.0 mmol), benzaldehyde (0.5 mmol), solvent (1.0 mL), CP catalyst, 25 °C.

b Yields are according to the analysis by ^1^H NMR: [moles of product per mol of aldehyde] × 100%; for details, see the SI.

When using catalyst **4**, the yield of 2-benzylidenemalononitrile increases from 43 to 99% on prolonging the time of reaction from 10 to 60 min (entries 1–6, Table 3; Figure S5). An influence of the amount of catalyst was investigated, resulting in an increase of the yield of product from 93 to >99% on augmenting the amount of catalyst from 1 to 2 mol % (Table 3, entries 6 and 7). In addition to methanol that appeared to be the optimum solvent, other common solvents were screened. In terms of product yields, the following trend can be made (Table 3, entries 6, 8–11): CH_3_OH (>99%) > H_2_O (98%) > C_2_H_5_OH (96%) > CH_3_CN (87%) > CH_3_Cl (67%). Remarkably, water is almost as good solvent as methanol, which is certainly an advantage of this catalytic system that can also operate under organic-solvent-free conditions. If compared to **4**, the CPs **1**–**3** and **5**–**8** show a somewhat lower activity with the yields of 2-benzylidenemalononitrile in the 90–98% interval (Table 3, entries 12–18). Blank tests indicate that the reaction between benzaldehyde and propanedinitrile (entries 19–21) is not effective without the catalyst (only 22% yield of product) or when applying as catalysts H_2_pdba (28% product yield) or CuCl_2_ (35% product yield). Besides, no side products were found in the reaction catalyzed by **4**, thus pointing out an excellent selectivity of this catalytic reaction. Although a connection between catalytic activity and structural characteristics of the catalyst may not be fully established in the present work, the superior activity of MOF **4** might be attributed to the existence of more easily accessible Lewis acid metal sites.^72,73^ In fact, both Cu(II) centers in this compound are 4-coordinate and display distorted square planar environments and the corresponding Lewis acid behavior.

Other benzaldehydes with functional groups (Table 4) and aromatic aldehydes (Table 5) were also screened for the substrate scope evaluation. These reactions were performed by reacting aldehyde substrates with propanedinitrile under optimum conditions (2.0 mol % **4**, 1 h, methanol solvent), and the product yields varied from 32 to 99% (Table 4). In particular, benzaldehyde substrates bearing substituents (−NO_2_, −Cl, −Br) with electron-withdrawing function are very reactive, allowing us to achieve product yields above 99% (entries 2–6, Table 4). This behavior is explained by an enhanced electrophilicity of the carbon center in the aldehyde substrate. However, the aldehydes containing an electron-donating substituent (−CH_3_, −OCH_3_, −OH) are less reactive and lead to inferior product yields (Table 4, entries 7 and 9).

**Table 4:** Substrate Scope in the Condensation Reaction between Substituted Benzaldehydes and Propanedinitrile Catalyzed by **4**^a^

entry	substituted benzaldehyde substrate (R-C_6_H_4_CHO)	product yield^b^ (%)
1	R = H	>99
2	R = 2-NO_2_	>99
3	R = 3-NO_2_	>99
4	R = 4-NO_2_	>99
5	R = 4-Cl	>99
6	R = 4-Br	>99
7	R = 4-CH_3_	98
8	R = 4-OCH_3_	79
9	R = 4-OH	32
10	cinnamaldehyde	79

a Reaction conditions: propanedinitrile (1.0 mmol), aldehyde (0.5 mmol), CH_3_OH (1.0 mL), catalyst 4 (2.0 mol %), 25 °C.

b Yields are according to the analysis by ^1^H NMR: [moles of product per mol of aldehyde substrate] × 100%.

**Table 5:** Knoevenagel Condensation of Aromatic Aldehydes with Propanedinitrile Catalyzed by **4**^a^

entry	substrate	product yield (%)
1	benzaldehyde	>99
2	1-naphthaldehyde	98
3	9-anthraldehyde	84

a Reaction conditions are similar to Table 4.

To check whether there is a correlation between the substrate size and the catalytic activity of **4** (Table 5), we compared 9-anthraldehyde (9.3 × 6.0 Å^2^) and 1-naphthaldehyde (7.0 × 5.9 Å^2^) with benzaldehyde (5.9 × 4.8 Å^2^).^68^ As expected, there is a slight decrease in product yield on increasing the molecular size of aromatic aldehyde from benzaldehyde (99% yield) to 1-naphthaldehyde (98% yield) and 9-anthraldehyde (84% yield). In the latter case, a larger size of 9-anthraldehyde may hamper its accessibility to Lewis acid centers.^74^

To access whether MOF **4** is stable throughout the catalytic cycle, experiments on recycling the catalyst were carried out. These revealed that **4** is active during at least five recycling runs (Figures S6 and S7, SI). Furthermore, PXRD data show that the structure of **4** is maintained after catalysis experiments, in spite of the appearance of further signals or broadening of some peaks. This type of changes might be explained by impurities or decline in the crystallinity after catalysis.

To confirm a heterogeneous character of the present Knoevenagel condensation reaction, the catalyst leaching test^75,76^ was undertaken. Hence, the control test was run in the presence of CP **4** before achieving an intermediate yield of the product (∼57% in 20 min). After this time, removal of the catalyst from the system was done via centrifugation, and the reaction continued for extra 40 min without the catalyst. As represented by the dotted line in Figure S5, the product yield does not change after the removal of the solid catalyst. These results confirm a heterogeneous character of the present transformation catalyzed by **4**. In an additional experiment after separating the catalyst, the filtered solution was dried in vacuo followed by the examination of the amount of copper. The performed analysis revealed only the traces of copper (0.035% of the catalyst amount used), thus indicating only insignificant leaching of copper from MOF 4.

In terms of the observed activity, catalyst **4** generally appears to be superior in the Knoevenagel condensation of aldehydes if compared to a number of reported coordination polymer catalysts (Table S5, SI).^5,50−52,67,77−83^ In particular, MOF **4** can lead to almost quantitative condensation of benzaldehyde with propanedinitrile with such advantages as a lower reaction temperature, an inferior loading of catalyst, and a shorter reaction time (Table S5).

Considering prior data for this type of catalytic transformations,^72,81^ a plausible mechanism for the Knoevenagel condensation catalyzed by **4** can be proposed (Scheme S1, SI). The unsaturated Cu(II) metal centers of the catalyst (4-coordinate copper centers) eventually act as the Lewis acid sites interacting with the H–C=O functionality of benzaldehyde, leading to its polarization and an enhanced electrophilicity of the corresponding C atom. Such polarization can facilitate a nucleophilic attack of this site by propanedinitrile acting as a nucleophile precursor. On the other hand, an interaction between the Lewis acid site and the −CN moiety of propanedinitrile augments an acidic character of the methylene functionality and enhances its deprotonation. The basic sites present in **4** (O-carboxylate sites) can easily abstract H^+^ from the −CH_2_– group to give rise to a nucleophile that would attack the H–C=O moiety of benzaldehyde and result in the carbon-carbon bond formation, followed by the dehydration to give the 2-benzylidenemalononitrile product.

## Conclusions

The present study highlighted the use of H_2_pdba (4,4′-(pyridine-3,5-diyl)dibenzoic acid) as a still little investigated pyridine-dicarboxylate linker source for generating coordination polymers. As a result, eight new CPs were hydrothermally assembled, isolated in good yields, and completely characterized. Structures and topologies in the metal–organic architectures of **1**–**8** were discussed with a focus on structural multiplicity. The majority of products are 2D coordination polymers, while compound **4** is a 3D metal–organic framework.

The catalytic activity of the prepared CPs was also evaluated in a reaction between benzaldehydes and propanedinitrile (Knoevenagel reaction), revealing a particularly promising behavior of MOF **4**. Apart from being recyclable, this heterogeneous catalyst shows an almost quantitative conversion of benzaldehyde into 2-benzylidenemalononitrile (>99% yield). Other benzaldehydes with electron-withdrawing groups are also reactive substrates in the present catalytic system.

In summary, this study provided new examples of functional coordination polymers that can be assembled by facile hydrothermal method using a blend of reaction conditions (autogenous pressure, temperature, and presence of crystallization mediators). The CPs prepared in the present work broadened a growing number of metal–organic networks assembled from still poorly explored pyridine-dicarboxylate linkers such as H_2_pdba and analogues. We expect that the current work can encourage additional research on the assembly of novel coordination polymers and on the search for their applications in heterogeneous catalysis and beyond.

## Supporting Information Available

Full synthetic procedures and analytical data for **1**–**8**; FTIR spectra (Figure S1); PXRD patterns (Figures S2 and S7); XPS spectrum (Figure S3); additional catalysis data (Figures S4–S7); structural parameters (Tables S1 and S2); reaction attempts for the synthesis of CPs (Table S3); porosity and gas sorption data (Table S4); reaction mechanism (Scheme S1); and comparison of catalytic activity (Table S5) (PDF)

## Accession Codes

CCDC-21674987–2167505 contain the supplementary crystallographic data for this paper. These data can be obtained free of charge via www.ccdc.cam.ac.uk/data_request/cif, by emailing data_request@ccdc.cam.ac.uk, or by contacting The Cambridge Crystallographic Data Centre, 12 Union Road, Cambridge CBZ, UK; fax: +44 1223 336033.
