# Supplementary material for: Coordination Polymers Constructed from an Adaptable Pyridine-Dicarboxylic Acid Linker: Assembly, Diversity of Structures, and Catalysis
Source: Inorg Chem. 2022 Nov 1;61(45):17951–62. doi: 10.1021/acs.inorgchem.2c01855 (PMC9775464; doi:10.1021/acs.inorgchem.2c01855)
Supplement: Supplementary file 1 — ic2c01855_si_001.pdf [file ic2c01855_si_001.pdf]

Supporting Information (SI)

Coordination Polymers Constructed from an Adaptable Pyridine-Dicarboxylic Acid Linker: Assembly, Diversity of Structures, and Catalysis

Xiaoyan Cheng,[†] Lirong Guo,*[,†] Hongyu Wang,[†] Jinzhong Gu,*[,†] Ying Yang,[†] Marina V. Kirillova,[‡] and Alexander M. Kirillov*[,‡]

[†]*State Key Laboratory of Applied Organic Chemistry, Key Laboratory of Nonferrous Metal Chemistry and Resources Utilization of Gansu Province, College of Chemistry and Chemical Engineering, Lanzhou University, Lanzhou 730000, People's Republic of China*

[‡]*Centro de Química Estrutural, Institute of Molecular Sciences, Departamento de Engenharia Química, Instituto Superior Técnico, Universidade de Lisboa, Av. Rovisco Pais, 1049-001, Lisbon, Portugal*

*To whom correspondence should be addressed. Tel.: +86-931-8915196; E-mail: gujzh@lzu.edu.cn; guolr@lzu.edu.cn; kirillov@tecnico.ulisboa.pt; Tel.: +351-218419396.

Contents

	General Methods	p. S2
	Synthesis and analytical data for **1–8**	p. S2
**Figure S1**	FTIR spectra of compounds **1–8**.	p. S4
**Figure S2**	PXRD patterns of compounds **1–8**	p. S6
**Figure S3**	X-ray photoelectron spectroscopy (XPS) spectrum of **6**.	p. S7
**Figure S4**	Typical ^{1}H NMR spectra of the reaction mixtures (and product yield calculation)	p. S8
**Figure S5**	Accumulation of product vs. time in the Knoevenagel condensation	p. S14
**Figure S6**	Catalyst recycling experiments	p. S14
**Figure S7**	PXRD patterns for **4**	p. S15
**Scheme S1**	Plausible mechanism for the Knoevenagel condensation reaction catalyzed by **4**	p. S15
**Table S1**	Selected bond lengths and angles for compounds **1–8**	p. S16
**Table S2**	Hydrogen bonds in crystal packing of **1–8**	p. S17
**Table S3**	Reaction attempts for the synthesis of CPs	p. S18
**Table S4**	Porosity and gas sorption data for **4**	p. S19
**Table S5**	Comparison of various catalysts in the Knoevenagel condensation of benzaldehyde and propanedinitrile	p. S20

General Methods. All chemicals and solvents were obtained from commercial suppliers. 4,4'-(Pyridine-3,5-diyl)dibenzoic acid (H_2pdba) was acquired from Jinan Henghua Sci. & Tec. Co., Ltd. C/N/H analyses were run on an Elementar Vario EL elemental analyzer. Bruker EQUINOX 55 spectrometer was used for recording the FTIR spectra (KBr discs). LINSEIS STA PT1600 thermal analyzer was used for the thermogravimetric (TGA) measurements (heating rate: 10˚C/min; N_2 flow). PXRD (powder X-ray diffraction) analyses were carried out on a Rigaku-Dmax 2400 diffractometer (Cu-Kα radiation, λ = 1.54060 Å). Solution ^{1}H NMR spectra were recorded on a JNM ECS 400M spectrometer.

Synthesis and Analytical Data for 1–8.

[Mn(μ$_4$-pdba)(H$_2$O)]$_n$ (1). A mixture of $MnCl_2·4H_2O$ (39.6 mg, 0.20 mmol), H_2pdba (63.8 mg, 0.20 mmol), NaOH (16.0 mg, 0.40 mmol), and H_2O (10 mL) was stirred at room temperature for 15 min, then sealed in a 25 mL Teflon-lined stainless steel vessel, and heated at 160 °C for 3 days, followed by cooling to room temperature at a rate of 10 °C·h^{-1}. Yellow block-shaped crystals of **1** were isolated manually, washed with distilled water and dried (yield 65% based on H_2pdba). Anal. Calcd for $C_{19}H_{13}MnNO_5$: C, 58.48; H, 3.36; N, 3.59. Found: C, 58.61; H, 3.38; N, 3.57%. FTIR (KBr, cm^{-1}): 3195 w, 3006 w, 2898 w, 2811 w, 1600 s, 1584m, 1546 s, 1439 w, 1394 s, 1332 w, 1217 w, 1196 w, 1109 w, 1014 w, 941 w, 891 w, 862 w, 813 w, 792 m, 747 w, 710w, 689 w, 648 w.

*{[M(μ$_3$-pdba)(phen)]·2H$_2$O}$_n$ (M = Co, **2**; Ni, **3**).* A mixture of $CoCl_2·6H_2O$ or $NiCl_2·6H_2O$ (0.20 mmol), H_2pdba (63.8 mg, 0.20 mmol), phen (40.0 mg, 0.20 mmol), NaOH (16 mg, 0.40 mmol), and H_2O (10 mL) was stirred at room temperature for 15 min, then sealed in a 25 mL Teflon-lined stainless steel vessel, and heated at 160 °C for 3 days, followed by cooling to room temperature at a rate of 10 °C·h^{-1}. Red (**2**) or green (**3**) block-shaped crystals of **2** and **3** were isolated manually, washed with distilled water and dried (yield 56% for **2**; 50% for **3**, based on H_2pdba). Anal. Calcd for $C_{31}H_{23}CoN_3O_6$ (**2**): C, 62.84; H, 3.91; N, 7.09. Found: C, 62.57; H, 3.93; N, 7.06%. FTIR (KBr, cm^{-1}): 3422 w, 3063 w, 1596 s, 1546s, 1514 w, 1411 s, 1394 s, 1320 w, 1225 w, 1163 w, 1101 w, 1019 w, 862w, 846 m, 817w, 788m, 730 w, 714 w, 677 w, 640 w. Anal. Calcd for $C_{31}H_{23}NiN_3O_6$ (**3**): C, 62.87; H, 3.91; N, 7.10. Found: C, 63.01; H, 3.90; N, 7.12%. FTIR (KBr, cm^{-1}): 3418 w, 3063 w, 1604 m, 1587 m, 1534m, 1514 w, 1423 s, 1378 s, 1320 w, 1237 w, 1167 w, 1101 w, 1014 w, 982w, 911 w, 871 w, 841w, 788m, 710 w, 668 w, 644 w.

{[Cu$_2$(μ$_3$-pdba)$_2$(bipy)]·2H$_2$O}$_n$ (4). A mixture of $CuCl_2·2H_2O$ (34.1 mg, 0.20 mmol), H_2pdba (63.8 mg, 0.20 mmol), 2,2′-bipy (31.2 mg, 0.20 mmol), NaOH (16 mg, 0.40 mmol), and H_2O (10 mL) was stirred at room

temperature for 15 min, then sealed in a 25 mL Teflon-lined stainless steel vessel, and heated at 160 °C for 3 days, followed by cooling to room temperature at a rate of 10 °C·h^{-1}. Blue block-shaped crystals of **4** were isolated manually, washed with distilled water and dried (yield 43% based on H_2pdba). Anal. Calcd for $C_{48}H_{34}Cu_2N_4O_{10}$: C, 60.44; H, 3.59; N, 5.87. Found: C, 60.23; H, 3.58; N, 5.89%. FTIR (KBr, cm^{-1}): 3459 w, 3075 w, 1608 s, 1554 w, 1443w, 1386 s, 1361 s, 1242 w, 1188 w, 1163 w, 1138 w, 1105 w, 1056 w, 1011 w, 920 w, 866 w, 817 w, 784 m, 771 w, 709 w, 668 w, 635 w.

*{[Co(μ_3-pdba)(bipy)]·2H$_2$O}$_n$ (**5**).* Synthesis of **5** was similar to **4** except using $CoCl_2·6H_2O$ (47.6 mg, 0.20 mmol) instead of $CuCl_2·2H_2O$. Pink block-shaped crystals of **5** were isolated manually, washed with distilled water and dried (yield 48% based on H_2pdba). Calcd for $C_{29}H_{23}CoN_3O_6$: C, 61.27; H, 4.08; N, 7.39%. Found: C, 61.53; H, 4.10; N, 7.36%. FTIR (KBr, cm^{-1}): 3422 w, 3067 w, 1608 s, 1546m, 1476 w, 1443m, 1414s, 1394 s, 1320 w, 1245 w, 1163 w, 1101 w, 1056 w, 1019 w, 911 w, 862 w, 821 w, 784 m, 771 w, 739 w, 714 w, 668 w, 627 w.

*[Co$_2$(μ_3-pdba)(μ-Hbiim)$_2$(Hbiim)]$_n$ (**6**).* A mixture of $CoCl_2·6H_2O$ (47.6 mg, 0.20 mmol), H_2pdba (63.8 mg, 0.20 mmol), H_2biim (26.8 mg, 0.20 mmol), NaOH (16.0 mg, 0.40 mmol), and H_2O (10 mL) was stirred at room temperature for 15 min, then sealed in a 25 mL Teflon-lined stainless steel vessel, and heated at 160 °C for 3 days, followed by cooling to room temperature at a rate of 10 °C·h^{-1}. Purple block-shaped crystals of **6** were obtained (yield 35% based on H_2pdba). Anal. Calcd for $C_{37}H_{26}Co_2N_{13}O_4$: C, 53.25; H, 3.14; N, 21.82. Found: C, 53.37; H, 3.17; N, 21.56. FTIR (KBr, cm^{-1}): 1637 m, 1620 w, 1587 w, 1551w, 1517 w, 1411 s, 1328 s, 1283 w, 1205 w, 1147m, 1085 w, 1014 w, 957 w, 940 w, 903 w, 850 w, 817 w, 780 m, 751 m, 697 w, 656 w.

*[M(μ_4-pdba)(py)]$_n$ (M = Co, 7; Ni, **8**).* A mixture of $CoCl_2·6H_2O$ or $NiCl_2·6H_2O$ (0.20 mmol), H_2pdba (63.8 mg, 0.20 mmol), py (0.50 mL, 6.20 mmol), and H_2O (10 mL) was stirred at room temperature for 15 min, then sealed in a 25 mL Teflon-lined stainless steel vessel, and heated at 160 °C for 3 days, followed by cooling to room temperature at a rate of 10 °C·h^{-1}. Pink (**7**) or green (**8**) block-shaped crystals of **7** and **8** were isolated manually, washed with distilled water and dried (yield 51% for **7**; 54% for **8**, based on H_2pdba). Anal. Calcd for $C_{24}H_{16}CoN_2O_4$ (**7**): C, 63.31; H, 3.54; N, 6.15. Found: C, 63.14; H, 3.53; N, 6.12%. FTIR (KBr, cm^{-1}): 1612 m, 1534w, 1418 s, 1386 s, 1332 w, 1217 w, 1167 w, 1072 w, 1010 w, 903w, 866 w, 812w, 788m, 755 w, 709 w, 693 w, 672 w, 631 w. Anal. Calcd for $C_{24}H_{16}NiN_2O_4$ (**8**): C, 63.34; H, 3.54; N, 6.16. Found: C, 63.21; H, 3.51; N, 6.19%. FTIR (KBr, cm^{-1}): 1612 s, 1583 m, 1558 w, 1534 m, 1427 s, 1386 s, 1332 w, 1216 w, 1167 w, 1072 w, 1014 w, 903w, 866 m, 812 w, 788m, 755 w, 709 w, 688 w, 627 w.

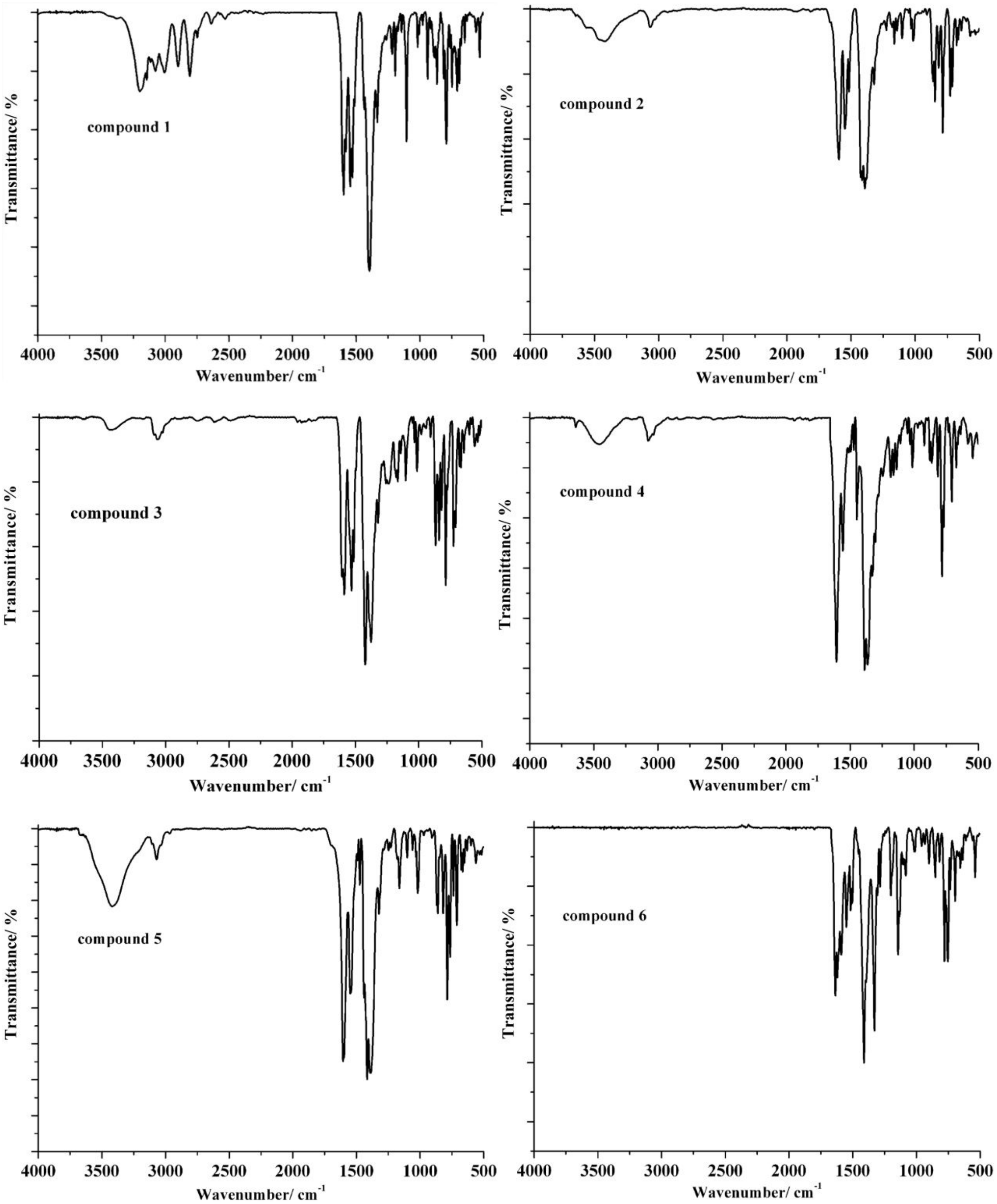
compound 1
compound 2
compound 3
compound 4
compound 5
compound 6
Transmittance/ %
Wavenumber/ cm^{-1}
4000
3500
3000
2500
2000
1500
1000
500

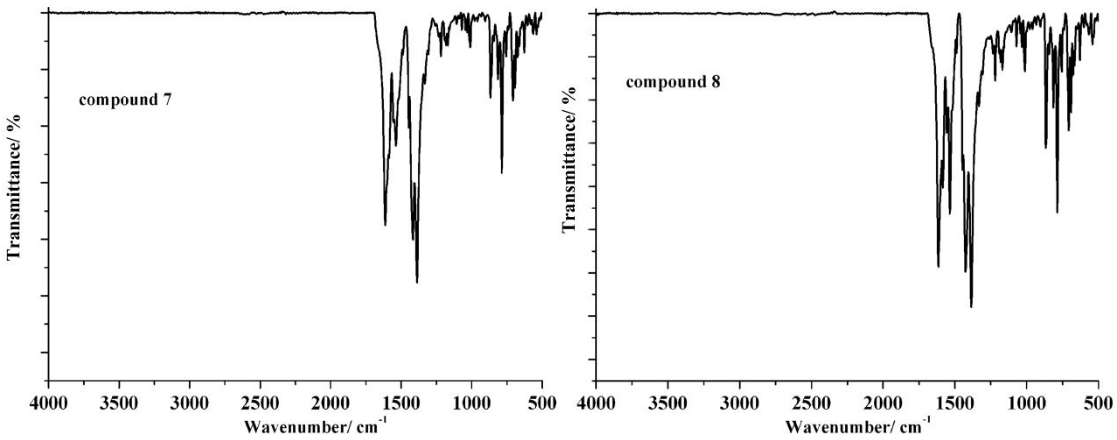

Figure S1. FTIR spectra of compounds **1–8**.

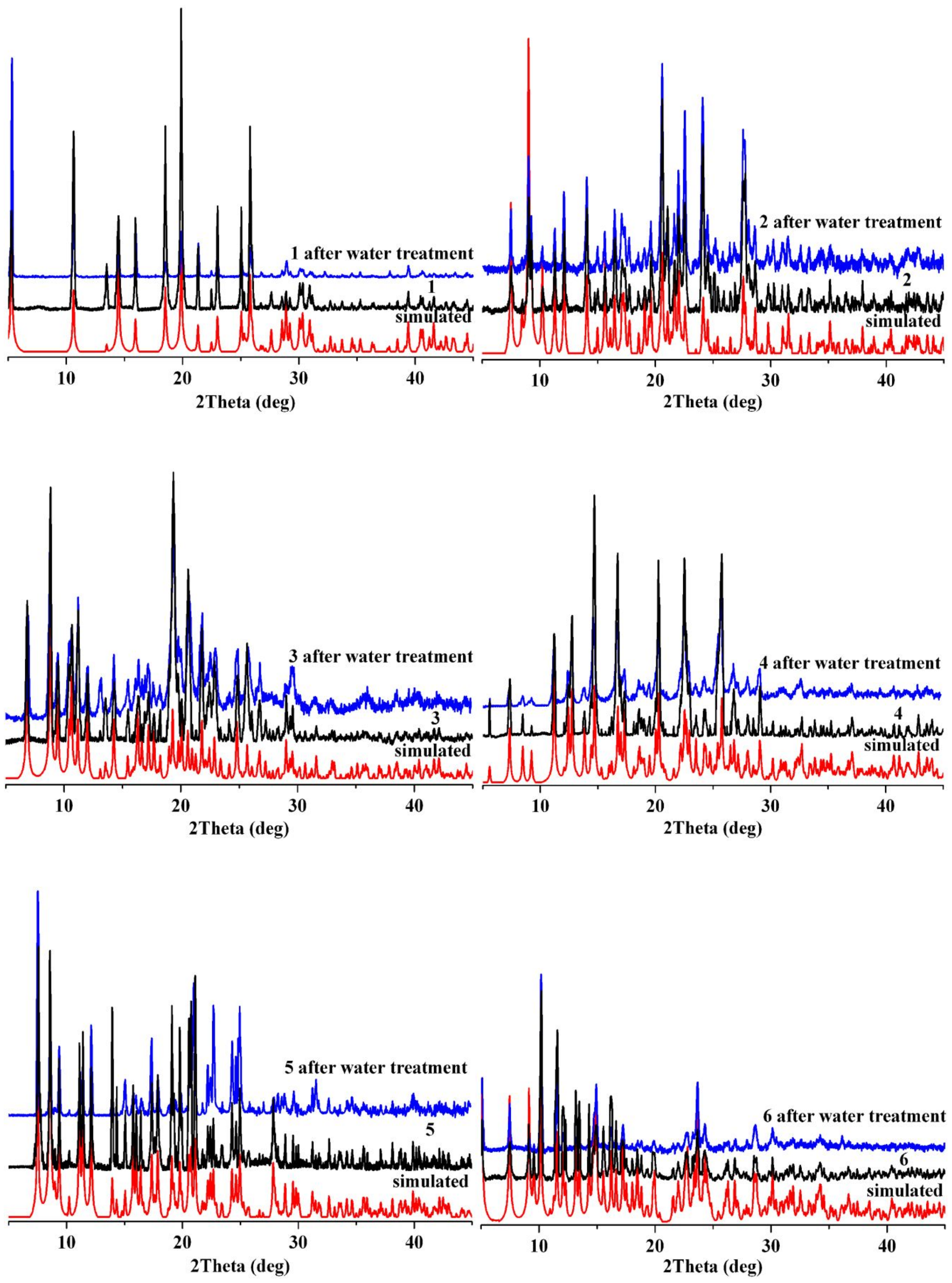
1 after water treatment
1
simulated
2 after water treatment
2
simulated
3 after water treatment
3
simulated
4 after water treatment
4
simulated
5 after water treatment
5
simulated
6 after water treatment
6
simulated
2Theta (deg)
10
20
30
40

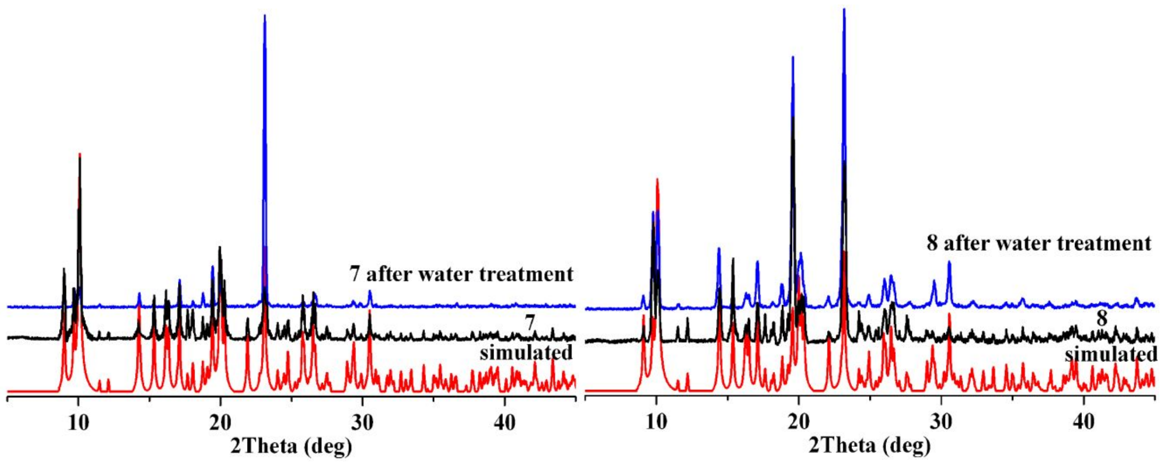

Figure S2. PXRD patterns of compounds **1**–**8** at room temperature. Black patterns correspond to the experimental data obtained using the as-synthesized bulk samples. Red patterns were simulated from the single crystal X-ray data (CIF files). Blue patterns are those after the water treatment experiment (the samples were kept in water at 50 ˚C for 12 h, then isolated and dried before PXRD measurements).

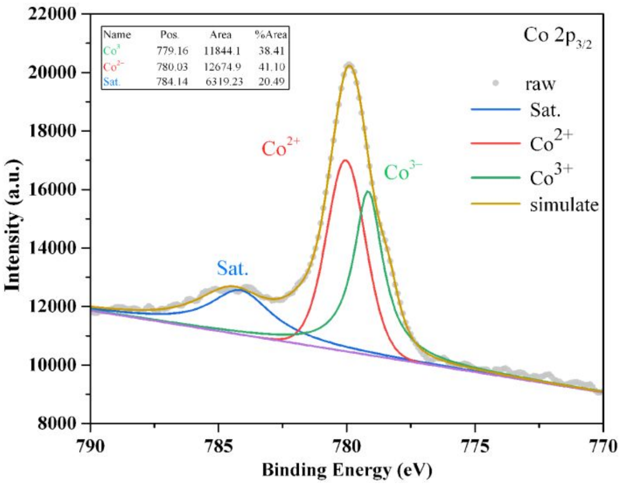

Figure S3. X-ray photoelectron spectroscopy (XPS) spectrum of **6**. The ratio of Co^{2+} and Co^{3+} peak areas is 1.07:1 (by peak deconvolution).

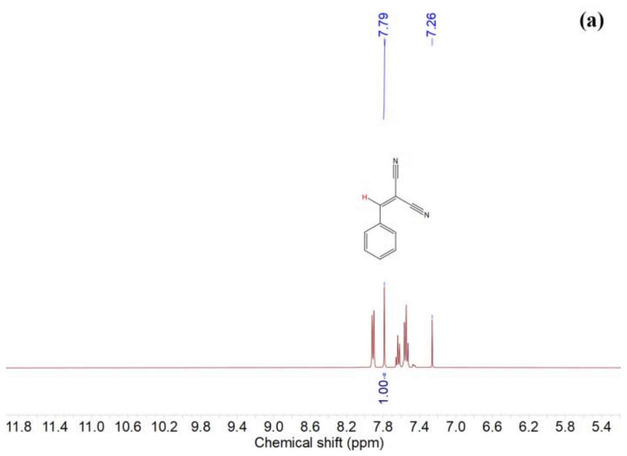
(a)
7.79
7.26
1.00
11.8 11.4 11.0 10.6 10.2 9.8 9.4 9.0 8.6 8.2 7.8 7.4 7.0 6.6 6.2 5.8 5.4
Chemical shift (ppm)

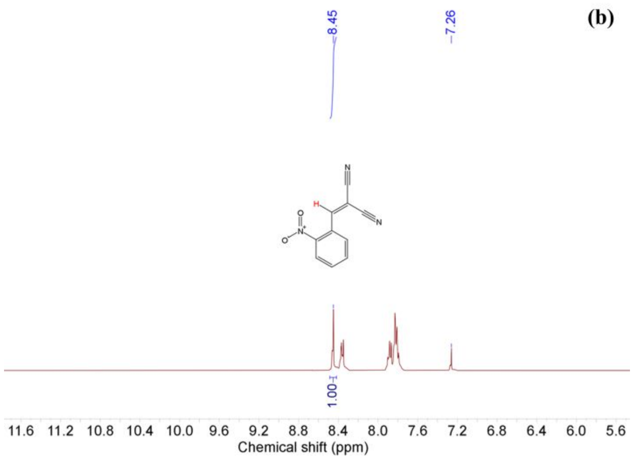
(b)
8.45
7.26
1.00
11.6 11.2 10.8 10.4 10.0 9.6 9.2 8.8 8.4 8.0 7.6 7.2 6.8 6.4 6.0 5.6
Chemical shift (ppm)

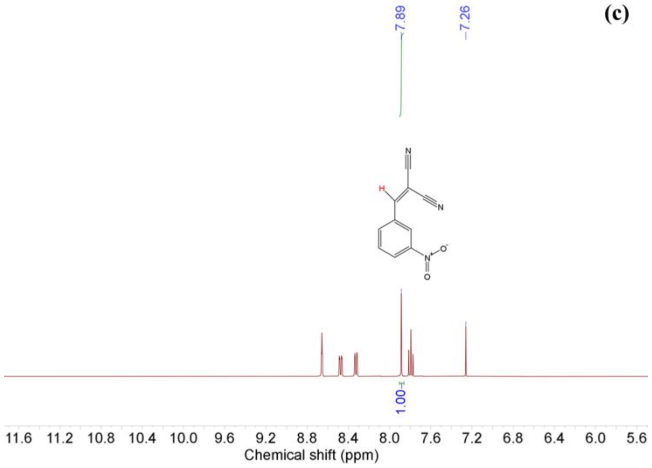
(c)
7.89
7.26
1.00
11.6 11.2 10.8 10.4 10.0 9.6 9.2 8.8 8.4 8.0 7.6 7.2 6.8 6.4 6.0 5.6
Chemical shift (ppm)

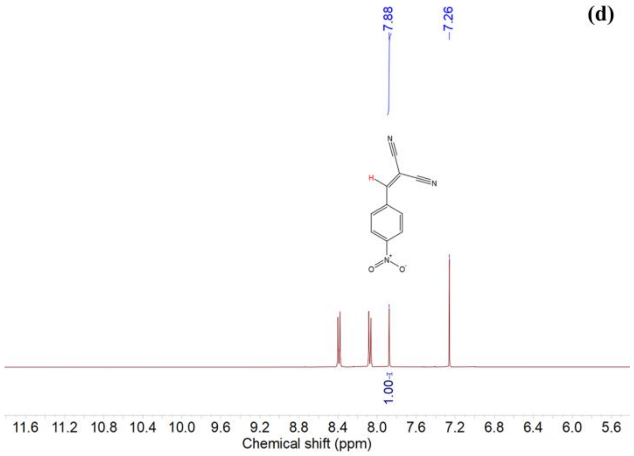
(d)
7.88
7.26
1.00
11.6 11.2 10.8 10.4 10.0 9.6 9.2 8.8 8.4 8.0 7.6 7.2 6.8 6.4 6.0 5.6
Chemical shift (ppm)

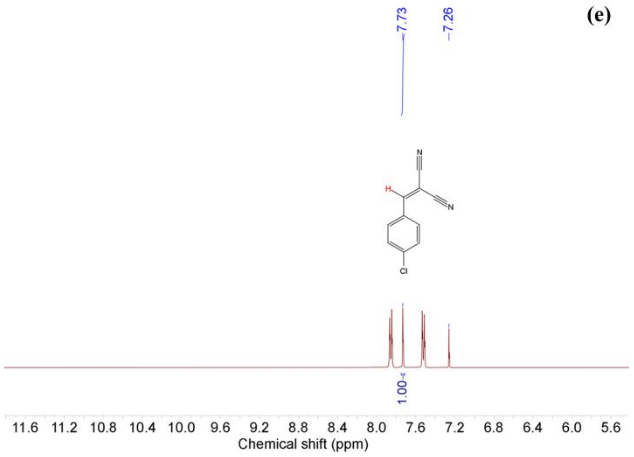
(e)
7.73
7.26
Cl
1.00
11.6 11.2 10.8 10.4 10.0 9.6 9.2 8.8 8.4 8.0 7.6 7.2 6.8 6.4 6.0 5.6
Chemical shift (ppm)

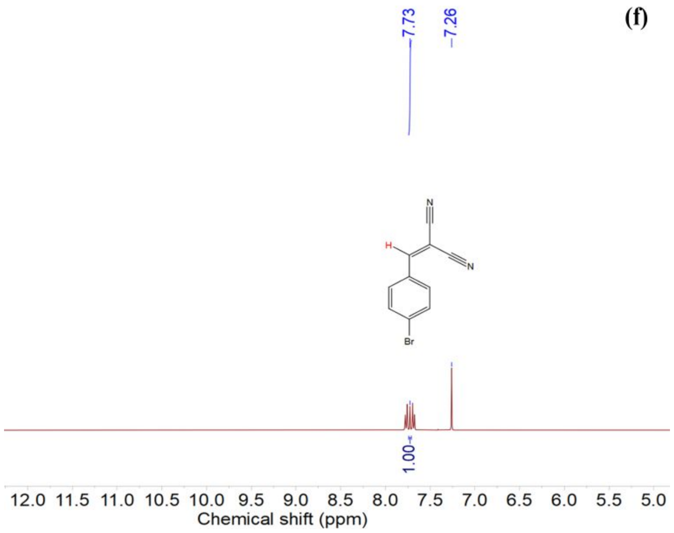
(f)
7.73
7.26
Br
1.00
12.0 11.5 11.0 10.5 10.0 9.5 9.0 8.5 8.0 7.5 7.0 6.5 6.0 5.5 5.0
Chemical shift (ppm)

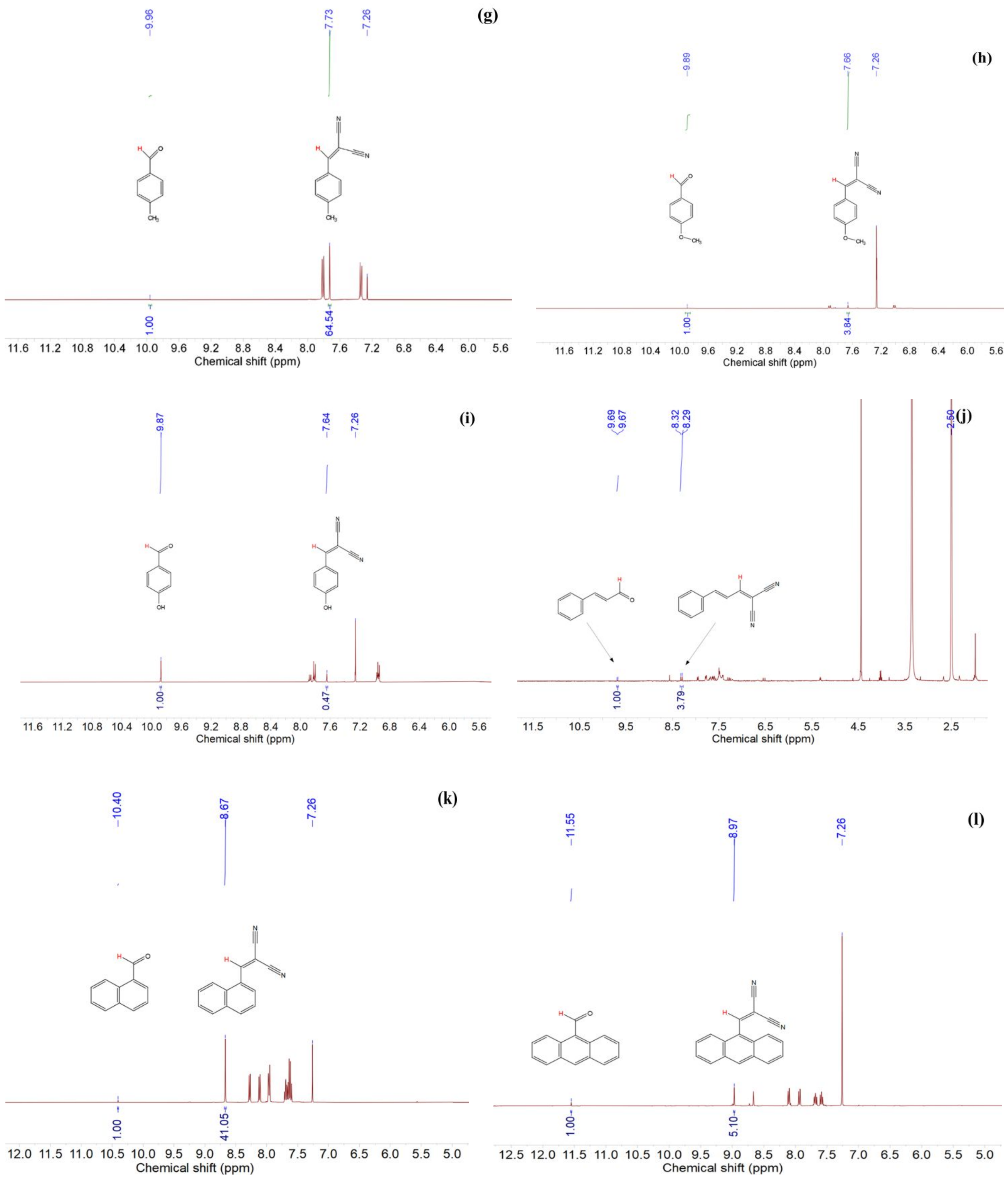

Figure S4. Examples for integration of ^{1}H NMR spectra for the determination of Knoevenagel condensation products. Reactions with different substrates (a)-(l). For details, see below the product yield calculations based on ^{1}H NMR.

Product Yield Calculations based on ^{1}H NMR (Figure S4)

(a): Conditions of Table 3, entry 6.

Product yield calculation in the Knoevenagel condensation reaction. The C(=O)*H* signal of benzaldehyde (substrate) appears at δ 10.02 ppm, while 2-benzylidenemalononitrile (product) shows a characteristic signal at δ 7.79 ppm.

Total integration of both signals: unreacted benzaldehyde + 2-benzylidenemalononitrile = 0 + 1.00 = 1.00.

Percentage of the unreacted substrate: 0/1.00 = 0%

Conversion of benzaldehyde = yield of 2-benzylidenemalononitrile = 100 − 0 = 100%.

(b) Conditions of Table 4, entry 2.

Product yield calculation in the Knoevenagel condensation reaction. The C(=O)*H* signal of 2-nitrobenzaldehyde (substrate) appears at δ 10.40 ppm, while (2-nitrobenzylidene)malononitrile (product) shows a characteristic signal at δ 8.45 ppm.

Total integration of both signals: unreacted 2-nitrobenzaldehyde + (2-nitrobenzylidene)malononitrile = 0 + 1.00 = 1.00.

Percentage of the unreacted substrate: 0/1.00 = 0%

Conversion of 2-nitrobenzaldehyde = yield of (2-nitrobenzylidene)malononitrile = 100−0 = 100%.

(c) Conditions of Table 4, entry 3.

Product yield calculation in the Knoevenagel condensation reaction. The C(=O)*H* signal of 3-nitrobenzaldehyde (substrate) appears at δ 10.03 ppm, while (3-nitrobenzylidene)malononitrile (product) shows a characteristic signal at δ 7.89 ppm.

Total integration of both signals: unreacted 3-nitrobenzaldehyde + (3-nitrobenzylidene)malononitrile = 0 + 1.00 = 1.00.

Percentage of the unreacted substrate: 0/1.00 = 0%

Conversion of 3-nitrobenzaldehyde = yield of (3-nitrobenzylidene)malononitrile = 100 − 0 = 100%.

(d) Conditions of Table 4, entry 4.

Product yield calculation in the Knoevenagel condensation reaction. The C(=O)*H* signal of 4-nitrobenzaldehyde (substrate) appears at δ 10.15 ppm, while (4-nitrobenzylidene)malononitrile (product) shows a characteristic signal at δ 7.88 ppm.

Total integration of both signals: unreacted 4-nitrobenzaldehyde + (4-nitrobenzylidene)malononitrile = 0 + 1.00 = 1.00.

Percentage of the unreacted substrate: 0/1.00 = 0%

Conversion of 4-nitrobenzaldehyde = yield of (4-nitrobenzylidene)malononitrile = 100−0 = 100%.

(e) Conditions of Table 4, entry 5.

Product yield calculation in the Knoevenagel condensation reaction. The C(=O)*H* signal of 4-chlorobenzaldehyde (substrate) appears at δ 9.97 ppm, while (4-chlorobenzylidene)malononitrile (product) shows a characteristic signal at δ 7.73 ppm.

Total integration of both signals: unreacted 4-chlorobenzaldehyde + (4-chlorobenzylidene)malononitrile = 0+1.00 = 1.00.

Percentage of the unreacted substrate: 0/1.00 = 0%

Conversion of 4-chlorobenzaldehyde = yield of (4-chlorobenzylidene)malononitrile = 100−0 = 100%.

(f) Conditions of Table 4, entry 6.

Product yield calculation in the Knoevenagel condensation reaction. The C(=O)*H* signal of 4-bromobenzaldehyde (substrate) appears at δ 9.97 ppm, while (4-bromobenzylidene)malononitrile (product) shows a characteristic signal at δ 7.73 ppm.

Total integration of both signals: unreacted 4-bromobenzaldehyde + (4-bromobenzylidene)malononitrile = 0+1.00 = 1.00.

Percentage of the unreacted substrate: 0/1.00 = 0%

Conversion of 4-bromobenzaldehyde = yield of (4-bromobenzylidene)malononitrile = 100−0 = 100%

(g) Conditions of Table 4, entry 7.

Product yield calculation in the Knoevenagel condensation reaction. The –CH peak of 4-methylbenzaldehyde (substrate) appears at 9.96 ppm while that of (4-methylbenzylidene)malononitrile (product) can be seen at 7.73 ppm.

Total amount: unreacted substrate (4-methylbenzaldehyde) + formed product (4-methylbenzylidene)malononitrile = 1+64.54 = 65.54

Percentage of the unreacted substrate: 1/65.54 = 1.53%

Conversion of 4-methylbenzaldehyde = yield of (4-methylbenzylidene)malononitrile = 100−1.53 = 98.5%.

(h) Conditions of Table 4, entry 8.

Product yield calculation in the Knoevenagel condensation reaction. The –CH peak of 4-methoxybenzaldehyde (substrate) appears at 9.89 ppm while that of (4-methoxybenzylidene)malononitrile (product) can be seen at 7.66 ppm.

Total amount: unreacted substrate (4-methoxybenzaldehyde) + formed product (4-methoxybenzylidene)malononitrile = 1 + 3.84 = 4.84

Percentage of the unreacted substrate: 1/4.84 = 20.7%

Conversion of 4-methoxybenzaldehyde = yield of (4-methoxybenzylidene)malononitrile = 100 − 20.7 = 79.3%.

(i) Conditions of Table 4, entry 9.

Product yield calculation in the Knoevenagel condensation reaction. The –CH peak of 4-hydroxybenzaldehyde (substrate) appears at 9.87 ppm while that of (4-hydroxybenzylidene)malononitrile (product) can be seen at 7.64 ppm.

Total amount: unreacted substrate (4-hydroxybenzaldehyde) + formed product (4-hydroxybenzylidene)malononitrile = 1+0.47 = 1.47

Percentage of the unreacted substrate: 1/1.47 = 68.0%

Conversion of 4-hydroxybenzaldehyde = yield of (4-hydroxybenzylidene)malononitrile = 100 − 68.0 = 32.0%.

(j) Conditions of Table 4, entry 10. d_6-DMSO solvent used.

Product yield calculation in the Knoevenagel condensation reaction. The –CH peaks of cinnamaldehyde (substrate) appear at 9.67 and 9.69 ppm while that of 2-(3-phenyl-2-propen-1-ylidene)propanedinitrile (product) can be seen at 8.29 and 8.32 ppm.

Total amount: unreacted substrate (cinnamaldehyde) + formed product 2-(3-phenyl-2-propen-1-ylidene)propanedinitrile = 1 + 3.79 = 4.79

Percentage of the unreacted substrate: 1/4.79 = 20.9%

Conversion of cinnamaldehyde = yield of 2-(3-phenyl-2-propen-1-ylidene)propanedinitrile = 100−20.9 = 79.1%.

(k) Conditions of Table 5, entry 2.

Product yield calculation in the Knoevenagel condensation reaction. The –CH peak of 1-naphthaldehyde (substrate) appears at 10.40 ppm while that of 2-(naphthalen-1-ylmethylidene)propanedinitrile (product) can be seen at 8.67 ppm.

Total amount: unreacted substrate (1-naphthaldehyde) + formed product 2-(naphthalen-1-ylmethylidene)propanedinitrile = 1 + 41.05 = 42.05

Percentage of the unreacted substrate: 1/42.05 = 2.4%

Conversion of 1-naphthaldehyde = yield of 2-(naphthalen-1-ylmethylidene)propanedinitrile = 100 − 2.4 = 97.6%.

(l) Conditions of Table 5, entry 3.

Product yield calculation in the Knoevenagel condensation reaction. The –CH peak of 9-anthraldehyde (substrate) appears at 11.55 ppm while that of 2-(anthracen-9-ylmethylene)malononitrile (product) can be seen at 8.97 ppm.

Total amount: unreacted substrate (9-anthraldehyde) + formed product 2-(anthracen-9-ylmethylene)malononitrile = 1+5.10=6.10

Percentage of the unreacted substrate: 1/6.10 = 16.4%

Conversion of 9-anthraldehyde = yield of 2-(anthracen-9-ylmethylene)malononitrile = 100 − 16.4 = 83.6%.

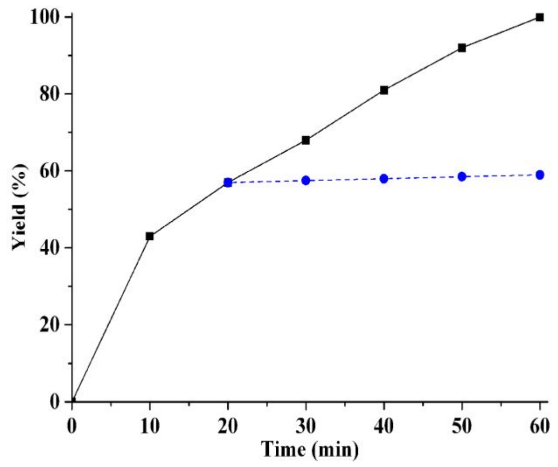

Figure S5. Accumulation of 2-benzylidenemalononitrile vs. time in the Knoevenagel condensation of benzaldehyde with propanedinitrile catalyzed by **4** (dotted line refers to the reaction after removal of the catalyst after 20 min reaction time; see text for details). Reaction conditions are those of Table 3, entries 1−6.

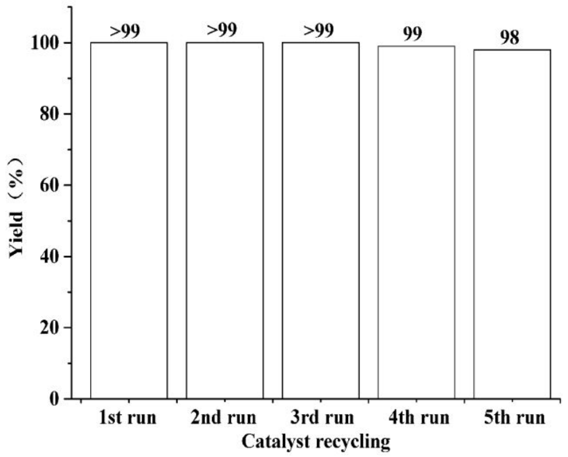

Figure S6. Catalyst recycling experiments in the Knoevenagel condensation of benzaldehyde with propanedinitrile catalyzed by **4**. Reaction conditions are those of Table 3, entry 6.

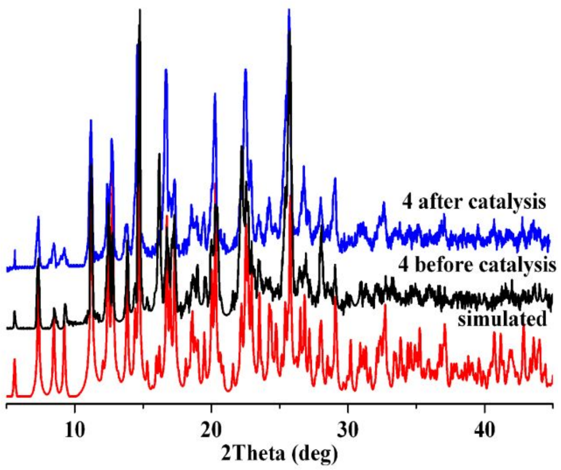

Figure S7. PXRD patterns for **4**: simulated (red), before (black) and after (blue) catalysis.

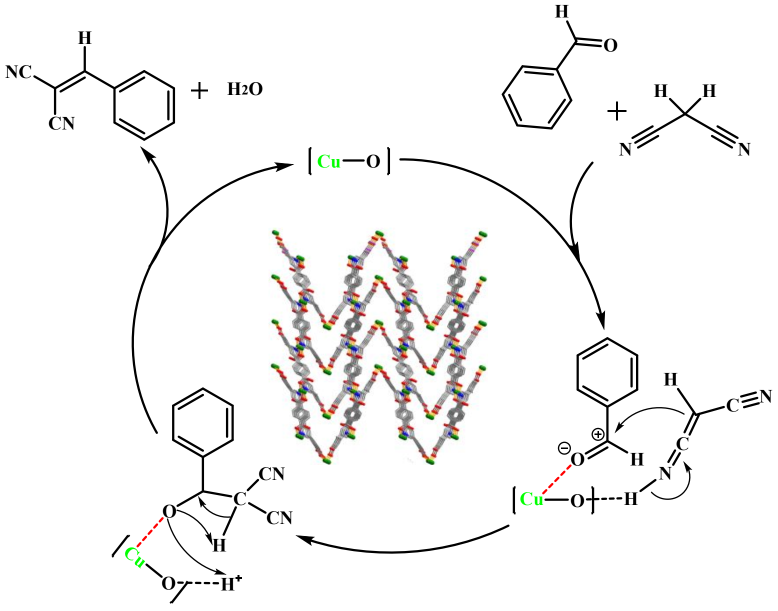

Scheme S1. Plausible mechanism for the Knoevenagel condensation reaction catalyzed by **4**.

Table S1. Selected Bond Lengths [Å] and Angles [°] for the Compounds **1**–**8**[a].

1					
Mn(1)-O(1)i	2.1896(15)	Mn(1)-O(1)ii	2.1896(15)	Mn(1)-O(2)	2.0815(14)
Mn(1)-O(2)iii	2.0815(14)	Mn(1)-O(3)	2.118(2)		
O(2)-Mn(1)-O(2)iii	143.69(9)	O(2)-Mn(1)-O(3)	108.15(5)	O(2)-Mn(1)-O(1)i	94.18(6)
O(2)-Mn(1)-O(1)ii	86.57(6)	O(1)i-Mn(1)-O(3)	88.80(3)	O(1)i-Mn(1)-O(1)ii	177.60(7)
2					
Co(1)-O(1)	2.027(3)	Co(1)-O(3)i	2.130(4)	Co(1)-O(4)i	2.296(4)
Co(1)-N(1)ii	2.139(4)	Co(1)-N(2)	2.134(4)	Co(1)-N(3)	2.169(4)
N(1)ii-Co(1)-N(3)	169.34(15)	O(4)i-Co(1)-N(1)	89.29(13)	N(1)ii-Co(1)-N(2)	97.18(15)
N(3)-Co(1)-N(2)	76.82(15)	O(4)i-Co(1)-N(2)	84.54(14)	O(4)i-Co(1)-N(3)	98.79(14)
O(1)-Co(1)-N(1)ii	93.67(14)	O(1)-Co(1)-N(2)	121.10(16)	O(1)-Co(1)-N(3)	82.23(14)
O(1)-Co(1)-O(3)i	94.75(16)	O(1)-Co(1)-O(4)i	153.46(15)	O(3)i-Co(1)-N(1)ii	100.44(14)
O(3)i-Co(1)-N(2)	138.71(15)	O(3)i-Co(1)-N(3)	89.74(14)	O(3)i-Co(1)-O(4)i	58.83(13)
3					
Ni(1)-O(1)	2.012(3)	Ni(1)-O(3)i	2.112(4)	Ni(1)-O(4)i	2.166(3)
Ni(1)-N(1)ii	2.107(4)	Ni(1)-N(2)	2.095(5)	Ni(1)-N(3)	2.079(5)
O(3)i-Ni(1)-O(1)	97.37(17)	O(4)i-Ni(1)-O(1)	158.76(17)	O(1)-Ni(1)-N(1)ii	99.34(16)
O(1)-Ni(1)-N(2)	91.17(17)	O(1)-Ni(1)-N(3)	101.10(18)	O(3)i-Ni(1)-O(4)i	62.14(13)
N(1)ii-Ni(1)-O(3)i	92.69(16)	N(1)ii-Ni(1)-O(4)i	87.83(13)	N(11)-Ni(1)-O(3)i	90.63(19)
O(4)i-Ni(1)-N(2)	83.97(15)	N(1)ii-Ni(1)-N(2)	168.47(17)	O(3)i-Ni(1)-N(3)	159.35(15)
O(4)i-Ni(1)-N(3)	98.38(15)	N(3)-Ni(1)-N(1)ii	93.35(19)	N(3)-Ni(1)-N(2)	79.9(2)
4					
Cu(1)-O(2)	1.932(2)	Cu(1)-O(7)i	1.930(2)	Cu(1)-N(3)	2.021(3)
Cu(1)-N(4)	2.012(3)	Cu(2)-N(1)	2.036(2)	Cu(2)-N(2)	2.032(2)
Cu(2)-O(3)ii	1.9181(18)	Cu(2)-O(6)iii	1.9261(18)		
O(2)-Cu(1)-O(7)i	98.46(10)	N(4)-Cu(1)-O(71)i	90.10(10)	O(2)-Cu(1)-N(4)	171.02(10)
N(3)-Cu(1)-O(7)i	166.04(12)	O(2)-Cu(1)-N(3)	91.22(10)	N(3)-Cu(1)-N(4)	80.84(11)
O(3)ii-Cu(2)-O(6)iii	176.41(11)	O(3)ii-Cu(2)-N(2)	88.39(9)	N(2)-Cu(2)-O(6)iii	92.06(8)
O(3)ii-Cu(2)-N(1)	92.78(9)	O(6)iii-Cu(2)-N(1)	87.16(9)	N(2)-Cu(2)-N(1)	173.70(11)
5					
Co(1)-O(2)i	2.030(3)	Co(1)-O(3)ii	2.289(3)	Co(1)-O(4)ii	2.130(3)
Co(1)-N(1)	2.134(3)	Co(1)-N(2)	2.137(3)	Co(1)-N(3)	2.117(3)
N(3)-Co(1)-O(2)i	115.65(13)	O(2)i-Co(1)-N(1)	95.71(11)	N(1)-Co(1)-N(3)	96.29(12)
O(2)i-Co(1)-O(4)ii	94.76(13)	O(4)ii-Co(1)-N(3)	145.59(11)	O(4)ii-Co(1)-N(1)	96.24(11)
O(2)i-Co(1)-N(2)	84.27(12)	N(2)-Co(1)-N(3)	76.62(12)	N(1)-Co(1)-N(2)	172.01(12)
N(2)-Co(1)-O(4)ii	91.73(11)	O(2)i-Co(1)-O(3)ii	153.82(13)	N(3)-Co(1)-O(3)ii	88.25(11)
N(1)-Co(1)-O(3)ii	91.84(10)	O(4)ii-Co(1)-O(3)ii	59.44(10)	N(2)-Co(1)-O(3)ii	91.63(11)
6					
Co(1)-O(2)	2.028(4)	Co(1)-O(4)i	2.083(4)	Co(1)-N(3)	2.086(4)
Co(1)-N(1)ii	2.273(4)	Co(1)-N(12)iii	2.142(4)	Co(2)-N(2)	1.921(4)
Co(2)-N(5)	1.939(5)	Co(2)-N(6)	1.940(4)	Co(2)-N(9)	1.926(4)
Co(2)-N(10)	1.916(5)	Co(2)-N(13)	1.918(4)		
O(2)-Co(1)-O(4)i	124.40(18)	O(2)-Co(1)-N(3)	98.43(16)	N(3)-Co(1)-O(4)i	136.4(2)
O(2)-Co(1)-N(12)iii	83.33(16)	O(4)i-Co(1)-N(12)iii	91.56(17)	N(3)-Co(1)-N(12)iii	102.18(16)
O(2)-Co(1)-N(1)ii	88.64(16)	O(4)i-Co(1)-N(1)ii	85.02(16)	N(3)-Co(1)-N(1)ii	88.49(16)
N(1)ii-Co(1)-N(12)iii	167.45(17)	N(10)-Co(2)-N(13)	82.48(18)	N(2)-Co(2)-N(10)	90.31(19)
N(2)-Co(2)-N(13)	92.57(18)	N(9)-Co(2)-N(10)	91.17(19)	N(9)-Co(2)-N(13)	91.17(18)

N(2)-Co(2)-N(9)	176.13(18)	N(5)-Co(2)-N(10)	171.49(19)	N(5)-Co(2)-N(13)	93.13(18)
N(2)-Co(2)-N(5)	82.57(18)	N(9)-Co(2)-N(5)	96.25(19)	N(6)-Co(2)-N(10)	91.25(19)
N(6)-Co(2)-N(13)	170.59(18)	N(2)-Co(2)-N(6)	94.48(18)	N(6)-Co(2)-N(9)	81.90(18)
N(6)-Co(2)-N(5)	93.94(19)				
**7**					
Co(1)-O(1)	2.0183(13)	Co(1)-O(2)i	2.1079(14)	Co(1)-O(3)ii	2.1807(13)
Co(1)-O(4)ii	2.1368(13)	Co(1)-N(1)iii	2.1569(15)	Co(1)-N(2)	2.1997(18)
O(1)-Co(1)-O(2)i	98.21(6)	O(1)-Co(1)-O(4)ii	164.62(6)	O(2)i-Co(1)-O(4)ii	87.88(5)
O(1)-Co(1)-N(1)iii	100.98(6)	O(2)i-Co(1)-N(1)iii	87.17(6)	O(4)ii-Co(1)-N(1)iii	93.39(5)
O(1)-Co(1)-O(3)ii	105.06(6)	O(2)i-Co(1)-O(3)ii	85.10(5)	O(3)ii-Co(1)-O(4)ii	61.19(5)
O(3)ii-Co(1)-N(1)iii	153.62(5)	O(1)-Co(1)-N(2)	84.25(6)	O(2)i-Co(1)-N(2)	175.35(6)
O(4)ii-Co(1)-N(2)	88.78(6)	N(1)iii-Co(1)-N(2)	96.26(6)	O(3)ii-Co(1)-N(2)	90.45(6)
**8**					
Ni(1)-O(1)	2.0053(13)	Ni(1)-O(2)i	2.0964(13)	Ni(1)-O(3)ii	2.1449(13)
Ni(1)-O(4)ii	2.1010(13)	Ni(1)-N(1)iii	2.0975(15)	Ni(1)-N(2)	2.1351(16)
O(1)-Ni(1)-O(2)i	97.31(6)	O(1)-Ni(1)-O(4)ii	163.90(6)	O(2)i-Ni(1)-O(4)ii	87.13(5)
O(1)-Ni(1)-N(1)iii	101.32(6)	O(2)i-Ni(1)-N(1)iii	87.20(6)	O(4)ii-Ni(1)-N(1)iii	94.33(6)
O(1)-Ni(1)-O(3)ii	102.68(6)	O(2)i-Ni(1)-O(3)ii	84.27(5)	O(3)ii-Ni(1)-O(4)ii	62.23(5)
O(3)ii-Ni(1)-N(1)iii	155.35(6)	O(1)-Ni(1)-N(2)	84.59(6)	O(2)i-Ni(1)-N(2)	175.25(6)
O(4)ii-Ni(1)-N(2)	89.89(6)	N(1)iii-Ni(1)-N(2)	96.72(6)	O(3)ii-Ni(1)-N(2)	91.07(6)

[a]Symmetry transformations used to generate equivalent atoms: i*x*, –*y*+1, *z*+1/2; ii –*x*+2, –*y*+1, –*z*+1; iii –*x*+2, *y*, –*z*+3/2 for **1**;i*x*+1, –*y*+3/2, *z*+3/2; ii *x*, *y*, *z*+1 for **2**; i*x*–1, –*y*+3/2, *z*–3/2; ii*x*, *y*, *z*–1 for **3**; I *x*–2, –*y*+1/2, *z*+1/2; ii –*x*+2, –*y*+1, –*z*; iii –*x*+3, –*y*, –*z* for **4**; i*x*, *y*, *z*–1; ii*x*+1, –*y*+1/2, *z*+1/2 for **5**; i –*x*+1, –*y*+1, –*z*; ii –*x*, –*y*+1/2, *z*+1/2; iii *x*, –*y*+3/2, *z*–1/2 for **6**; i –*x*, –*y*+1, –*z*+3; ii *x*–1, *y*–1, *z*+2; iii *x*–1, *y*, *z*+1 for **7** and **8**.

Table S2. Hydrogen Bonds in Crystal Packing [Å, °] of **1**, **4** and **6**.

Complexes	D-H…A	*d*(D-H)	*d*(H…A)	*d*(D…A)	∠DHA	Symmetry code
**1**	O(3)-H(1W)···O(1)	0.861	2.202	2.689	115.65	-*x*+2, *y*+1, -*z*+3/2
	O(3)-H(2W)···O(1)	0.861	2.004	2.689	135.80	*x*, *y*+1, *z*
**4**	O(9)-H(1W)···O(8)	0.850	2.209	2.825	129.22	*x*-2, -*y*+1/2, *z*+1/2
	O(9)-H(2W)···O(2)	0.850	2.368	3.025	134.4	
	O(10)-H(3W)···O(1)	0.850	2.026	2.811	153.14	*x*+1, *y*+1, *z*
	O(10)-H(4W)···O(5)	0.850	2.143	2.974	165.52	*x*-1, *y*+1, *z*
**6**	N(4)-H(1)···O(2)	0.860	2.105	2.851	144.78	
	N(7)-H(2)···N(8)	0.860	1.986	2.795	156.42	-*x*, -*y*+1, -*z*+2
	N(11)-H(8)···O(3)	0.860	2.188	2.816	129.66	-*x*+1, *y*+1/2, -*z*+1/2

Table S3. Reaction Attempts for the Synthesis of CPs.[a]

No	Metal(II) precursor	Crystallization mediator (CM)	M^{2+}/H_2pdba/ CM/NaOH molar ratio	Result	Product formula (when available)
1	$CuCl_2·2H_2O$	-	1/1/-/2	no crystallization	-
2	$CoCl_2·6H_2O$	-	1/1/-/2	no crystallization	-
3	$NiCl_2·6H_2O$	-	1/1/-/2	no crystallization	-
4	$MnCl_2·4H_2O$	-	1/1/-/2	monocrystals/X-ray structure	[Mn(μ_4-pdba)(H_2O)]$_n$ (**1**)
5	$ZnCl_2$	-	1/1/-/2	no crystallization	-
6	$CdCl_2·H_2O$	-	1/1/-/2	no crystallization	-
7	$CuCl_2·2H_2O$	phen	1/1/1/2	no crystallization	-
8	$CoCl_2·6H_2O$	phen	1/1/1/2	monocrystals/X-ray structure	{[Co(μ_3-pdba)(phen)]·$2H_2O$}$_n$ (**2**)
9	$NiCl_2·6H_2O$	phen	1/1/1/2	monocrystals/X-ray structure	{[Ni(μ_3-pdba)(phen)]·$2H_2O$}$_n$(**3**)
10	$MnCl_2·4H_2O$	phen	1/1/1/2	no crystallization	-
11	$ZnCl_2$	phen	1/1/1/2	polycrystals/not suitable for SCXD	-
12	$CdCl_2·H_2O$	phen	1/1/1/2	no crystallization	-
13	$CuCl_2·2H_2O$	2,2′-bipy	1/1/1/2	monocrystals/X-ray structure	{[Cu_2(μ_3-pdba)$_2$(bipy)]·$2H_2O$}$_n$ (**4**)
14	$CoCl_2·6H_2O$	2,2′-bipy	1/1/1/2	monocrystals/X-ray structure	{[Co(μ_3-pdba)(bipy)]·$2H_2O$}$_n$ (**5**)
15	$NiCl_2·6H_2O$	2,2′-bipy	1/1/1/2	polycrystals/not suitable for SCXD	-
16	$MnCl_2·4H_2O$	2,2′-bipy	1/1/1/2	no crystallization	-
17	$ZnCl_2$	2,2′-bipy	1/1/1/2	no crystallization	-
18	$CdCl_2·H_2O$	2,2′-bipy	1/1/1/2	no crystallization	-
19	$CuCl_2·2H_2O$	4,4′-bipy	1/1/1/2	no crystallization	-
20	$CoCl_2·6H_2O$	4,4′-bipy	1/1/1/2	no crystallization	-
21	$NiCl_2·6H_2O$	4,4′-bipy	1/1/1/2	no crystallization	-
22	$MnCl_2·4H_2O$	4,4′-bipy	1/1/1/2	no crystallization	-
23	$ZnCl_2$	4,4′-bipy	1/1/1/2	no crystallization	-
24	$CdCl_2·H_2O$	4,4′-bipy	1/1/1/2	no crystallization	-
25	$CuCl_2·2H_2O$	H_2biim	1/1/1/2	no crystallization	-
26	$CoCl_2·6H_2O$	H_2biim	1/1/1/2	monocrystals/X-ray structure	[Co_2(μ_3-pdba)(μ-Hbiim)$_2$(H_2biim)]$_n$ (**6**)
27	$NiCl_2·6H_2O$	H_2biim	1/1/1/2	no crystallization	-
28	$MnCl_2·4H_2O$	H_2biim	1/1/1/2	no crystallization	-
29	$ZnCl_2$	H_2biim	1/1/1/2	no crystallization	-

30	$CdCl_2 \cdot H_2O$	H_2biim	1/1/1/2	no crystallization	-
31	$CuCl_2 \cdot 2H_2O$	py	1/1/31/	no crystallization	-
32	$CoCl_2 \cdot 6H_2O$	py	1/1/31/	monocrystals/X-ray structure	[Co(μ_4-pdba)(py)]$_n$ (**7**)
33	$NiCl_2 \cdot 6H_2O$	py	1/1/31/	monocrystals/X-ray structure	[Ni(μ_4-pdba)(py)]$_n$ (**8**)
34	$MnCl_2 \cdot 4H_2O$	py	1/1/31/	no crystallization	-
35	$ZnCl_2$	py	1/1/31/	no crystallization	-
36	$CdCl_2 \cdot H_2O$	py	1/1/31/	no crystallization	-

[a]Hydrothermal synthesis: stainless steel reactor (Teflon-lined, 25 mL volume), water as solvent (10 mL), 160 ˚C, 3 days.

Table S4. Porosity and Gas Sorption Data for Compound **4**.[a]

Compound	Void volume (%)	Amount of adsorbed N_2 (cm^3/g)	BET surface area (m^2/g)
**4**	5.0	2.1	1.0

[a]Prior to gas adsorption tests, the samples were activated to remove the guest water molecules (samples were heated under vacuum at 120 ˚C for 18 h). The sorption measurements were carried out on the activated samples and the N_2 adsorption/desorption isotherms were measured at 77 K.

Table S5. Comparison of Various Catalysts for the Knoevenagel Condensation Reaction between Benzaldehyde and Propanedinitrile.

Entry	Catalyst	Catalyst (mol%)	Solvent	Time (h)	Temp. (°C)	Conversion (%)	Ref.
1	$\{[Cu_2(\mu_3\text{-pdba})_2(bipy)]\cdot 2H_2O\}_n$	2	MeOH	1	RT	>99	This work
2	$Zn_3(OH)(ATTCA)_2(H_2O)]\cdot C_2H_6NH_2\cdot 4DMF\cdot H_2O$	10	DCM	5	RT	94	77
3	$[Zn_3(L)_2(\mu_2\text{-}OH)_2]_n$	4	H_2O	8	90	78	78
4	$[Zn_2(TCA)(BIB)_{2.5}]\cdot(NO_3)$	0.3	–	1	60	99	52
5	$\{[Zr_6(\mu_3\text{-}O)_4(\mu_3\text{-}OH)_4(OH)_4(H_2O)_4(DCBA)_2]\cdot 5DMF\cdot 3H_2O\}$	10	DCM	5	RT	89	5
6	$\{[Ba_3Zn_4(TDP)_2(HCO_2)_2(OH_2)_2]\cdot 7DMF\cdot 4H_2O\}_n$	3	Ethanol	1	60	99	67
7	$\{[Zn(L)(bpfp)](H_2O)_3\}_n$	5	DMF	3	35	98	50
8	$[Zn(L)(H_2O)_2]_n\cdot n$(N-methylformamide)]	3	MeOH	1.5	40	75	79
9	$[PbL_2]\cdot 2DMF\cdot 6H_2O$	3	CH_3CN	24	RT	>99	80
10	$[Co_2(tdc)_2(tpxn)]\cdot 6H_2O\}_n$	2	MeOH	1	25-30	96	81
11	$[Zn(L1)(NMeF)]_n\cdot n(NMeF)$	3	H_2O	6	50	>99	82
12	$\{[Cd(Py_2TTz)(2\text{-}NH_2\text{-}BDC)]\cdot(DMF)\cdot 0.5(H_2O)\}_n$	2	–	6	60	99.8	51
13	$[Zn_{15}(mbpz)_6(Hmbpz)_6(L\text{-}NO_2)_4(HL\text{-}NO_2)_2(\mu_3\text{-}OH)_2]_n$	0.6	–	1	80	>99	83

Linkers in coordination polymer catalysts: H_3ATTCA: 2-amino[1,1:3,1-terphenyl]-4,4,5-tricarboxylic acid; H_2L: 2-(hydroxymethyl)-1H-benzo[d]imidazole-5-carboxylic acid; H_3TCA: tricarboxytriphenyl amine, BIB: 1,3-bis(imidazol-1-ylmethyl)benzene; H_4DCBA: 4'',6'-diamino-5',5''-bis(4-carboxyphenyl)-[1,1':3',1'':3'',1'''-quaterphenyl]-4,4'''-dicarboxylic acid; H_6TDP: 2,4,6- tri(2,4-dicarboxyphenyl)pyridine; H_2L: methyl-3-hydroxy-5-carboxy-2-thiophenecarboxylate, bpfp:

bis(4-pyridylformyl)piperazine; L: 5-acetamidoisophthalic acid; HL: *N*-(4-carboxyphenyl)isonicotinamide 1-oxide; tpxn: N,N'-(1,4-phenylenebis(methylene))bis(1-(pyridin-2-yl)-N-(pyridin-2-ylmethyl)methanamine, H_2tdc: thiophene-2,5-dicarboxylic acid; H_2L1: 5-{(pyren-4-ylmethyl)amino}isophthalic acid; 2-NH_2-H_2BDC: 2-amino-1,4-benzenedicarboxylic acid, Py2TTz: 2,5-bis(4-pyridyl)thiazolo[5,4-d]thiazole; H_2mbpz: 3,3',5,5'-Tetramethyl-4,4'-bi-1H-pyrazole, H_2L-NO_2: 2,2'-Dinitro-[1,1'-biphenyl]-4,4'-dicarboxylic acid.
